# Acquisition of functions on the outer capsid surface during evolution of double-stranded RNA fungal viruses

**DOI:** 10.1371/journal.ppat.1006755

**Published:** 2017-12-08

**Authors:** Carlos P. Mata, Daniel Luque, Josué Gómez-Blanco, Javier M. Rodríguez, José M. González, Nobuhiro Suzuki, Said A. Ghabrial, José L. Carrascosa, Benes L. Trus, José R. Castón

**Affiliations:** 1 Department of Structure of Macromolecules, Centro Nacional de Biotecnología (CNB-CSIC), Campus Cantoblanco, Madrid, Spain; 2 Centro Nacional de Microbiología/ISCIII, Majadahonda, Madrid, Spain; 3 Institute of Plant Science and Resources, Okayama, Japan; 4 Department of Plant Pathology, University of Kentucky, Lexington, KY, United States of America; 5 Imaging Sciences Laboratory, CIT, NIH, Bethesda, MD, United States of America; Indiana University Bloomington, UNITED STATES

## Abstract

Unlike their counterparts in bacterial and higher eukaryotic hosts, most fungal viruses are transmitted intracellularly and lack an extracellular phase. Here we determined the cryo-EM structure at 3.7 Å resolution of Rosellinia necatrix quadrivirus 1 (RnQV1), a fungal double-stranded (ds)RNA virus. RnQV1, the type species of the family *Quadriviridae*, has a multipartite genome consisting of four monocistronic segments. Whereas most dsRNA virus capsids are based on dimers of a single protein, the ~450-Å-diameter, T = 1 RnQV1 capsid is built of P2 and P4 protein heterodimers, each with more than 1000 residues. Despite a lack of sequence similarity between the two proteins, they have a similar α-helical domain, the structural signature shared with the lineage of the dsRNA bluetongue virus-like viruses. Domain insertions in P2 and P4 preferential sites provide additional functions at the capsid outer surface, probably related to enzyme activity. The P2 insertion has a fold similar to that of gelsolin and profilin, two actin-binding proteins with a function in cytoskeleton metabolism, whereas the P4 insertion suggests protease activity involved in cleavage of the P2 383-residue C-terminal region, absent in the mature viral particle. Our results indicate that the intimate virus-fungus partnership has altered the capsid genome-protective and/or receptor-binding functions. Fungal virus evolution has tended to allocate enzyme activities to the virus capsid outer surface.

## Author summary

Most fungal RNA viruses are transmitted by cytoplasmic interchange without leaving the host. We report the cryo-electron microscopy structure, at near-atomic resolution, of the double-stranded RNA Rosellinia necatrix quadrivirus 1 (RnQV1); this virus infects the fungus *Rosellinia necatrix*, a pathogenic ascomycete to a wide range of plants. At difference most dsRNA viruses, whose capsid is made of protein homodimers, RnQV1 is based on a single-shelled lattice built of 60 P2-P4 heterodimers. Despite a lack of sequence similarity, P2 and P4 have a similar α-helical domain, a structural signature shared with the dsRNA virus lineage. In addition to organizing the viral genome and replicative machinery, P2 and P4 have acquired new functions by inserting complex domains in preferential insertion sites. Whereas the P2 insertion domain has a fold like that of actin-binding proteins, the structure of the P4 insertion domain indicates proteolytic activity. Understanding the structure of a fungal virus capsid with enzyme activities could allow its development as nanoreactors for biotechnological application.

## Introduction

The virus capsid is sufficiently robust to protect the genome and ensure its propagation during passage from one host cell or organism to another; it must simultaneously be labile enough to allow genome delivery within the host cell to initiate infection. The capsid is not only a metastable macromolecular assembly, but is also a dynamic structure whose protein components have transient conformations related to its distinct roles during the viral life cycle [1, 2]. Numerous high resolution structural studies have shown that, despite the vast diversity and complexity of virus capsids, assembly of these closed protein shells is limited to a few capsid protein (CP) folds. As a result, evolution leads to a small number of viral lineages that share a CP fold as well as a set of common assembly principles [3].

Icosahedral viruses are grouped in four lineages, (i) the dsDNA viruses with an upright double β-barrel CP (prototypes are phage PRD1 and adenovirus), (ii) the tailed dsDNA phages, tailed haloarchaeal viruses, and herpesviruses, which all share the Hong Kong 97 (HK97)-like CP fold, (iii) the ssRNA picornavirus-like superfamily with a single β-barrel as the CP fold, and (iv) the dsRNA or bluetongue virus (BTV)-like viruses [4]. The dsRNA viruses have a specialized icosahedral capsid that remains structurally undisturbed during endogenous transcription, thus avoiding induction of host cell defense mechanisms [5]. The capsid also participates in genome transcription and replication by organizing packaged genomes and the RNA-dependent RNA polymerase (RdRp) complex(es). This unusual T = 1 capsid is built from 60 asymmetrical dimers of a single CP (i.e., a 120-subunit T = 1 capsid), in which the CP fold is the hallmark of the BTV lineage. This capsid structure is found in members of the families *Reoviridae* [6–9], *Picobirnaviridae* [10] and *Cystoviridae* [11, 12], and in the mycoviruses of the families *Totiviridae* [13–15], *Partitiviridae* [16, 17], and *Megabirnaviridae* [18]. Chrysoviruses, a group of dsRNA mycoviruses with a multipartite genome, have a T = 1 capsid with 60 subunits of a single 982-amino-acid CP, but the CP is an almost perfect structural duplication of a single domain [19–21]. Birnaviruses are an exception, as they have a single T = 13 shell and lack the T = 1 capsid [22, 23].

A distinctive feature of the fungal dsRNA viruses is their lack of an extracellular route of transmission [24]. Probably as a result of their intracellular transmission (by cell division, sporogenesis and cytoplasmic fusion), most mycoviruses have a single-shell capsid. In contrast, reo- and cystoviruses have multilayered concentric capsids, with one or two T = 13 shells surrounding the T = 1 layer (referred to as the inner core).

Whereas most dsRNA virus capsids are based on dimers of a single protein, the type species of the family *Quadriviridae*, Rosellinia necatrix Quadrivirus 1 (RnQV1), has a single-shelled T = 1 capsid formed by 60 heterodimers of two proteins of 1356 (P2) and 1059 residues (P4) [25]. RnQV1 is a mycovirus with a multipartite genome consisting of four monocistronic dsRNA segments (genome sizes range from 3.7 to 4.9 kbp), in which one or two dsRNA segments are encapsidated in a similar particle [26]. dsRNA-1 codes for a protein of unknown function, dsRNA-2 encodes the P2 CP, dsRNA-3 codes for RdRp (1117 amino acids), and dsRNA-4 codes for the P4 CP. In RnQV1 strain W1075, P2 and P4 are cleaved into several discrete peptides without altering capsid structural integrity, whereas in strain W1118 both proteins remain nearly intact [27, 28]. Here we report the three-dimensional (3D) cryo-EM structure of the RnQV1 W1118 capsid at 3.7 Å resolution. We found that P2-P4 heterodimers are organized in a quaternary structure similar to that of reovirus, chrysovirus and totivirus. Despite the low sequence identity (~15%, after introduction of 23% gaps), superimposition of P2 and P4 showed a common core similar to the CP fold of the dsRNA virus lineage. P2 and P4 have also acquired new functions by insertion of complex domains with potential enzyme activity in preferential insertion sites.

## Results

### Near-atomic resolution cryo-EM structure of RnQV1-W1118

**Fig 1:**
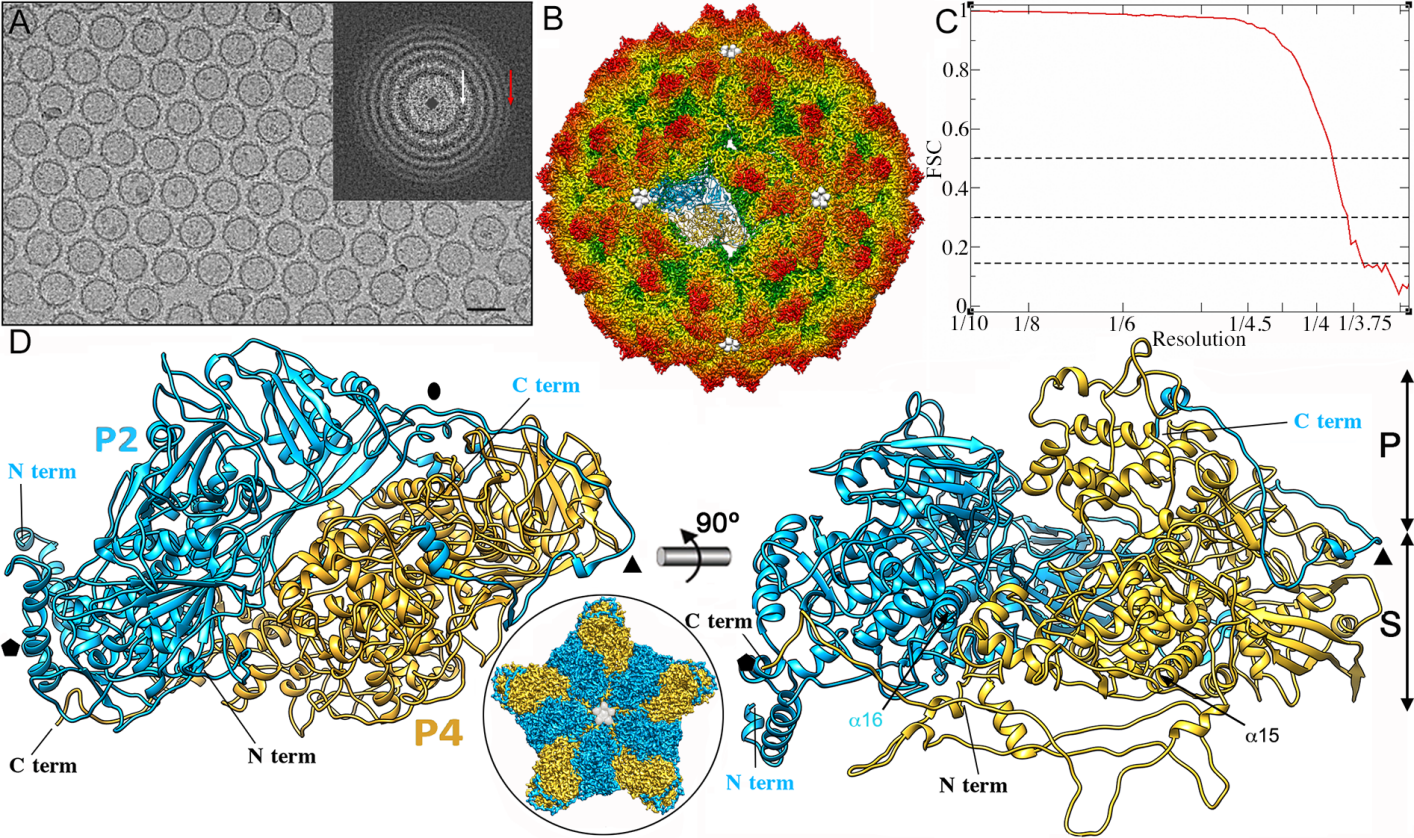
Three-dimensional cryo-EM reconstruction of RnQV1 virions at 3.7 Å resolution. (A) Cryo-EM image of RnQV1. Bar = 500 Å. Inset, power spectrum of a motion-corrected, averaged image. The first Thon ring is at 8.7 Å (white arrow) and the highest visible Thon ring is at 3.4 Å resolution (red arrow). (B) Radially color-coded atomic model of the RnQV1 capsid viewed along a two-fold axis. The atomic structures of a dimer of P2 (blue) and P4 (yellow) are shown. (C) Fourier shell correlation (FSC) resolution curve for RnQV1. Resolution based on the 0.5, 0.3 and 0.143 criteria is 3.88, 3.80 and 3.70 Å respectively. (D) Ribbon diagrams of P2 (blue) and P4 (yellow) (top view, left; side view, right). N and C termini and CP domains (shell, S; protruding, P) are indicated. Symbols indicate icosahedral symmetry axes. Inset, atomic model of the RnQV1 capsid pentamer viewed along an icosahedral five-fold axis, showing the five P2 (blue) and P4 (yellow) proteins.

**Fig 2:**
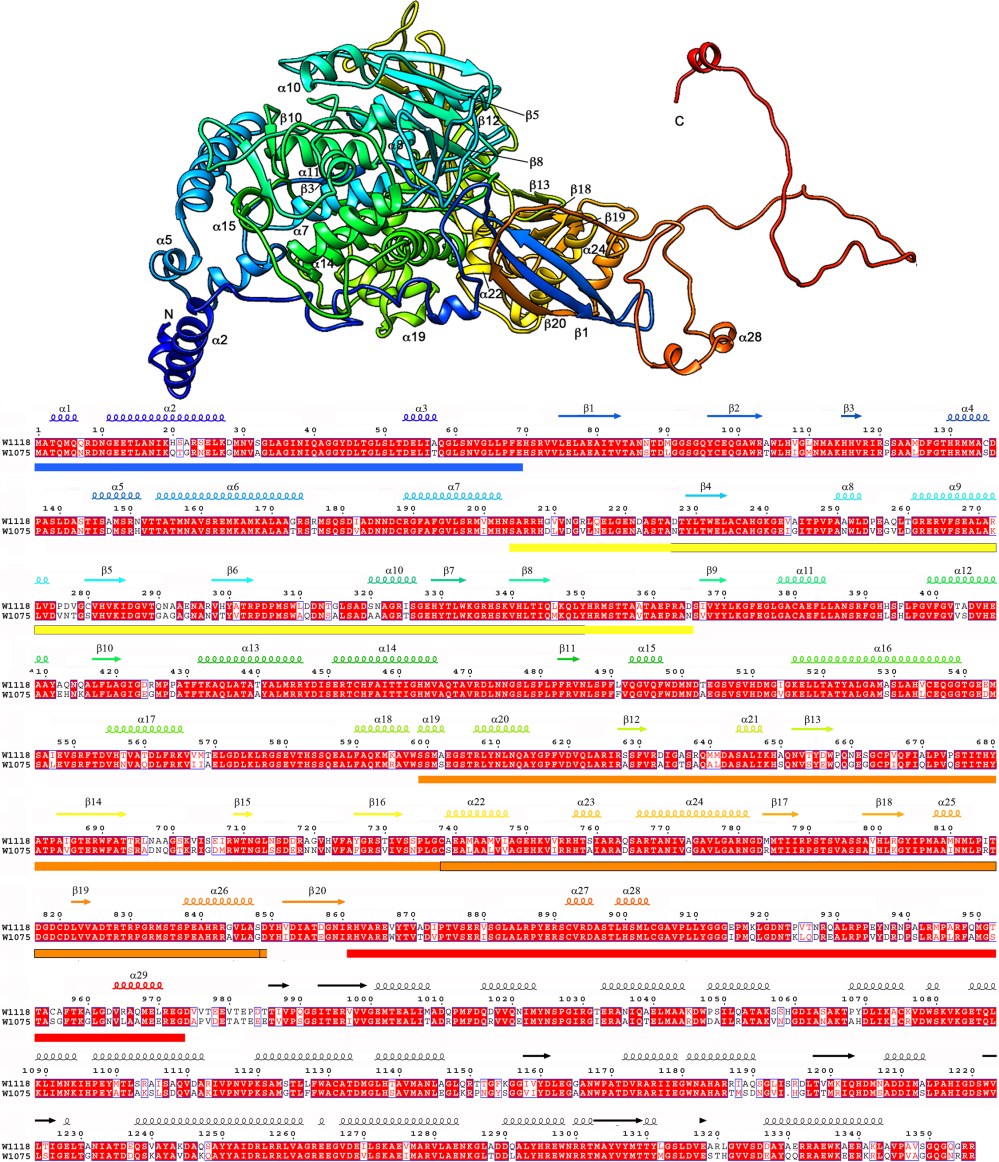
Atomic model of P2. Top, ribbon diagram of the RnQV1 W1118 P2 capsid protein (side view), rainbow-colored from blue (N terminus) to red (C terminus). The N and C termini are indicated. Bottom, sequence and SSE analysis of the 1356-residue P2, rainbow-colored from blue (N terminus) to red (C terminus). The C-terminal 383-residue segment is not present; the consensus-predicted SSE for P2 (black) are shown. Colored boxes indicate insertions in the conserved domain (SID or P2*; defined in Fig 4) at the N terminus (blue), middle (yellow and orange) and C terminus (red). The boxed yellow region (segment 227–350) indicates the gelsolin-like fold; boxed orange region (segment 738–848) indicates the SIID. The aligned P2 sequences for W1118 and W1075 are shown.

**Fig 3:**
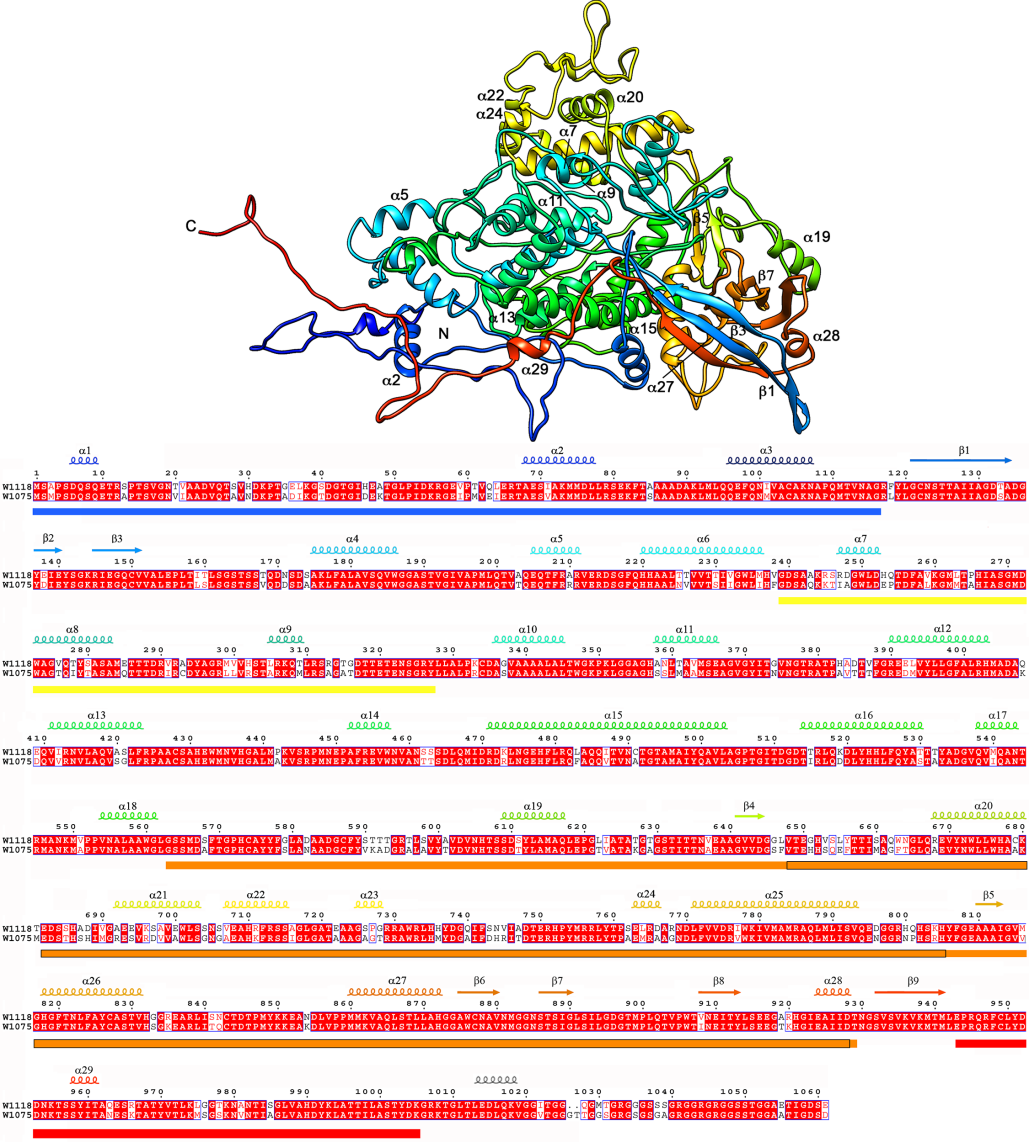
Atomic model of P4. Top, ribbon diagram of the RnQV1 W1118 P4 capsid protein (side view), rainbow-colored from blue (N terminus) to red (C terminus). The N and C termini are indicated. Bottom, sequence and SSE analysis of the 1059-residue P4, rainbow-colored from blue (N terminus) to red (C terminus). The C-terminal 54-residue segment is not visible; the consensus-predicted SSE for P2 (black) is shown. Colored boxes indicate insertions in the conserved domain (SID or P4*; defined in Fig 4) at the N terminus (blue), middle (yellow and orange) and C-terminus (red). The boxed orange regions, segments 648–805 and 817–929, indicate the putative protease-like fold and SIID, respectively. The aligned P4 sequences for W1118 and W1075 are shown.

Purified empty RnQV1-W1118 particles were visualized in a 300-kV FEI Titan Krios cryo-electron microscope (Fig 1A). Signal was detectable to 3.4 Å (Fig 1A inset, red arrow). We merged ~37,500 particle images to calculate a 3.7 Å resolution map (Fig 1B), as estimated by the criterion of 0.143 Fourier shell correlation coefficient (Fig 1C). Some regions on the uneven outer surface of the RnQV1 capsid, especially those at the five-fold axis, were less well-defined than its smooth inner surface (S1A Fig), although the backbone was traceable unambiguously (S1B Fig). Approximately 96% of the amino acid side chains were resolved (S1C and S1D Fig). We used the Coot program to build the polypeptide chain, based initially on structural predictions for some amino acid sequence segments (Figs 2 and 3).

The ~470 Å-diameter T = 1 capsid shows 12 outwardly protruding decamers at the pentameric positions (Fig 1B, orange), each bearing five copies of P2 and P4 [25]. Each pentameric capsomer is formed by an inner ring of five P2 monomers (Fig 1D, blue; inset) surrounded by an outer ring of five partially intercalated P4 monomers (Fig 1D, yellow; inset), in which the asymmetric unit is a P2-P4 heterodimer (Fig 1B and 1D). The quaternary organization of P2 and P4 in the capsid shell is similar to that of homodimers of reoviruses and other dsRNA mycoviruses.

Our near-atomic models for P2 and P4 had 972 and 1005 residues, respectively, of the total 1356 (P2) and 1059 (P4) amino acids. P2 lacks 383 residues in the C-terminal region, predicted to have a high helical content (Fig 2), and P4 lacks 54 C-terminal residues (Fig 3). The last visible P4 residue is Lys1005 (Fig 3), located at the five-fold axis channel. Although the remaining P4 C-terminal 54-residue segment is very flexible (see below), five small densities that follow Lys1005 occlude the channel (Fig 1B, white).

The P2 383-residue C-terminal region is invisible in our map. Analysis of this region in the RnQV1 capsid 3D reconstruction at lower resolution indicated no density that could account for the missing P2 domain. Sequence-based SSE prediction methods indicated a large number of α-helices, which are predicted very reliably. To determine whether the P2 C-terminal region was present, we first analyzed the purified RnQV1 capsids by SDS-PAGE. P4 and P2 proteins, detected as 110- and 100-kDa bands as reported [25], were analyzed by MALDI-TOF/TOF mass spectrometry (MS). Mass spectra showed no evidence of P2 C-terminal peptides (S2 Fig and S1 Table), nor of the P4 55-residue C-terminal region. With previous biochemical analyses [25] and structural studies of this region (see below), these data suggested that the P2 C-terminal end is indeed proteolyzed and absent from the viral particles.

The structure of P2 (built from 29 α-helices and 20 β-strands) and P4 (29 α-helices, 9 β-strands) was organized similarly and was divided into two domains, a shell (S) and a protruding (P) domain (Fig 1D, right). Although the P4 (residues 648–805) and P2 (204–365) protruding domains were similar in size, they had very different folds. P2 and P4 overlapped considerably at the S domain. Both had a long α-helix tangential to the capsid surface, the α16 helix in P2 (36 Å long, 24 residues) and α15 in P4 (50 Å, 33 residues), a common characteristic of the dsRNA virus lineage CP (Fig 1D, right, arrows).

#### Structural comparison of the RnQV1 capsid proteins

The RnQV1 capsid is built from 60 heterodimers; this differs from most dsRNA virus 120-subunit capsids with 60 copies of a homodimer. The 60-subunit chrysovirus T = 1 capsid is a partial exception, as each subunit has two domains with the same fold.

**Fig 4:**
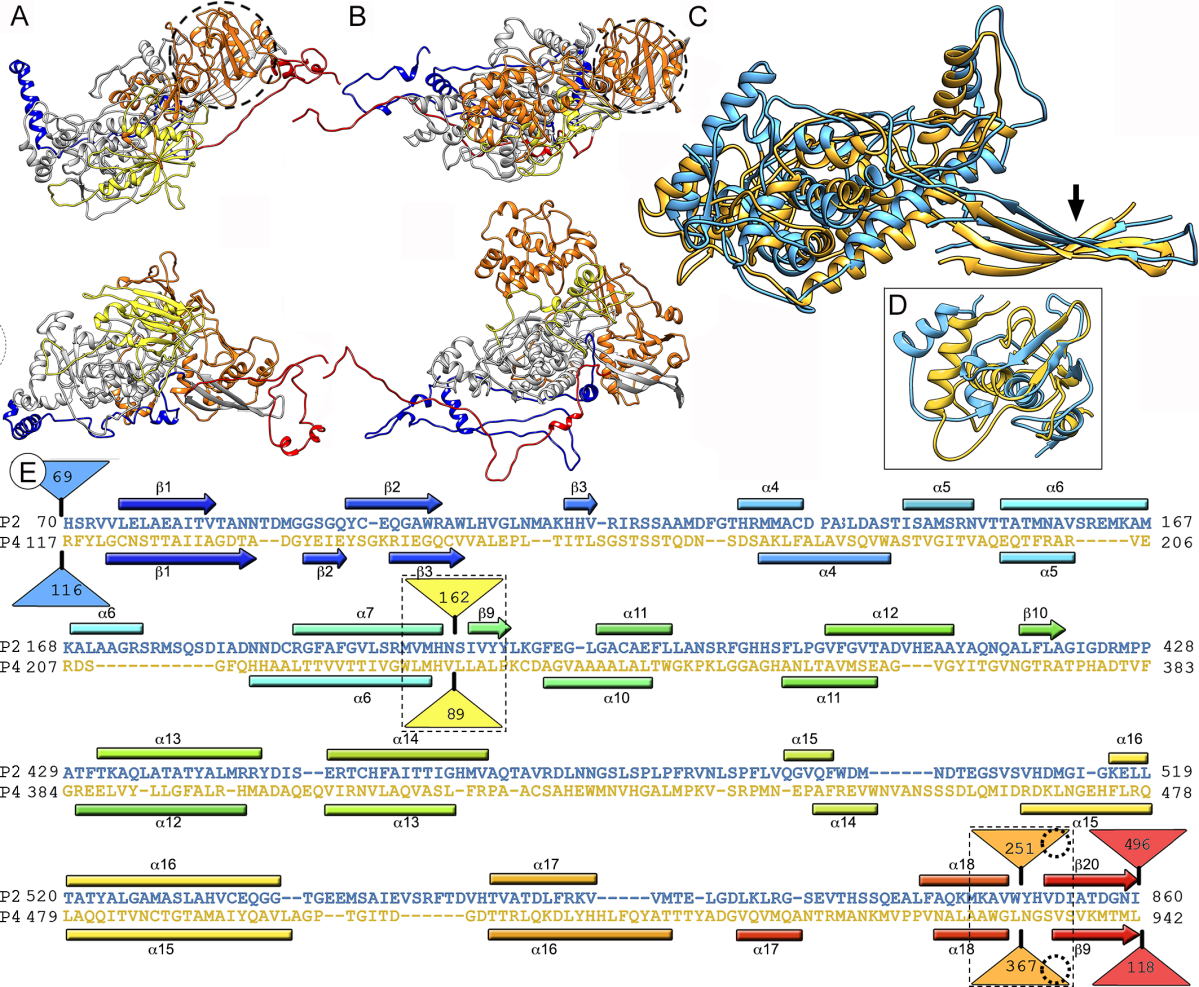
Structural similarities of P2 and P4 capsid proteins. (A, B) Atomic models of P2 (A, top view, top; side view, bottom) and P4 (B, top view, top; side view, bottom). Conserved domains are grey (SID of P2 and P4, or P2* and P4*); insertions at the N terminus (blue), middle (yellow and orange), and C terminus (red) are also shown (the last 54 residues of P2 are omitted). Dashed circles correspond to P2 and P4 SIID. (C) Superimposed P2* (blue) and P4* (yellow) (non-superimposable regions for both CP are omitted). Arrow indicates shared β-sheet. (D) The P2 Cys738-Ser848 and the P4 Gly817-Thr929 segments (SIID of P2 and P4), included in the middle insertion (orange), are structurally aligned. (E) Sequence alignment of P2* (blue) and P4* (yellow) resulting from Dali structural alignment. α-helices (rectangles) and β-strands (arrows) are rainbow-colored from blue (N terminus) to red (C terminus) for each protein. Dashed rectangles indicate favorable insertion sites, triangles represent non-aligned segments (sizes indicated), and dashed circles correspond to P2 and P4 SIID. Insertions in sites 1 and 2 are shown as yellow and orange triangles, respectively.

RnQV1 heterodimers resemble the homodimers in reo-, cysto- and totivirus, in which the asymmetric dimer is organized with the longitudinal axis of each protomer in parallel. Structural alignment of dsRNA virus CP has been used successfully to show common folds [3, 29]. Although they have no sequence similarity, P2 and P4 are morphologically similar (Fig 4A and 4B) and were aligned using the Dali server. Alignment parameters showed a root mean square deviation (rmsd) of 5.5 Å for 539 superposed Cα (z-score = 7.0), indicating a common fold (Table 1). In the S domain (Fig 4A and 4B, grey), 11 α-helices and three β-strands (which form a β-sheet) matched very well and required only minor adjustment [referred to as shell I domain (SID); Fig 4C, S1 Movie]. For simplicity, P2 and P4 SID are termed P2* and P4* in Table 1.

**Table 1:** Dali-based structural comparisons.

Input structures	rmsd	z-score	Aligned residues
P2 vs P4	5.5	7.0	539
P2* vs P4*	5.1	12.0	295
P2* vs PcV domain A	5.2	8.1	227
P4* vs PcV domain A	6.4	8.1	250
P2* vs L-A Gag	4.8	10.2	233
P4* vs L-A Gag	4.9	9.9	256
P2* vs PcV domain B	4.7	6.3	230
P4* vs PcV domain B	5.1	5.7	219

Located on the conserved β-sheet of P2* and P4* (Fig 4C, arrow), P2 (residues 738–848) and P4 (817–929) have another equivalent region, denoted shell II domain (SIID), which faces the outer capsid surface (Fig 4A and 4B, dashed circles). P2 and P4 SIID have three α-helices and three β-strands each that were also matched using the Chimera MatchMaker routine (Fig 4D; S3 Fig and S2 Movie). SID and SIID are described separately, as only SID is shared with the dsRNA virus lineage fold (see below). The 13 α-helices and 6 β-strands common to P2 and P4 constitute most of the shell region. Equivalent secondary structure elements (SSE) had the same polarity, and β-sheets were formed by the same number of β-strands.

In addition to the N- and C-terminal insertions (as large as 496 residues at the P2 C terminus), we detected two favorable insertion sites on the P2 and P4 outer surfaces, in which variations had been introduced by insertion of segments from 89 to 367 residues (Fig 4E, dashed rectangles). The first insertion site (insertion site 1) is located downstream of α-helices 7 and 6 of P2 and P4, respectively (Fig 4E, yellow triangles). The second site (site 2) has insertions of 251 or 367 residues for P2 and P4, respectively, and contains the SIID; P2 and P4 SIID precede β-strands 20 and 9, respectively (Fig 4E, dashed circles in orange triangles).

### Putative enzymatic domains on the RnQV1 capsid outer surface

**Fig 5:**
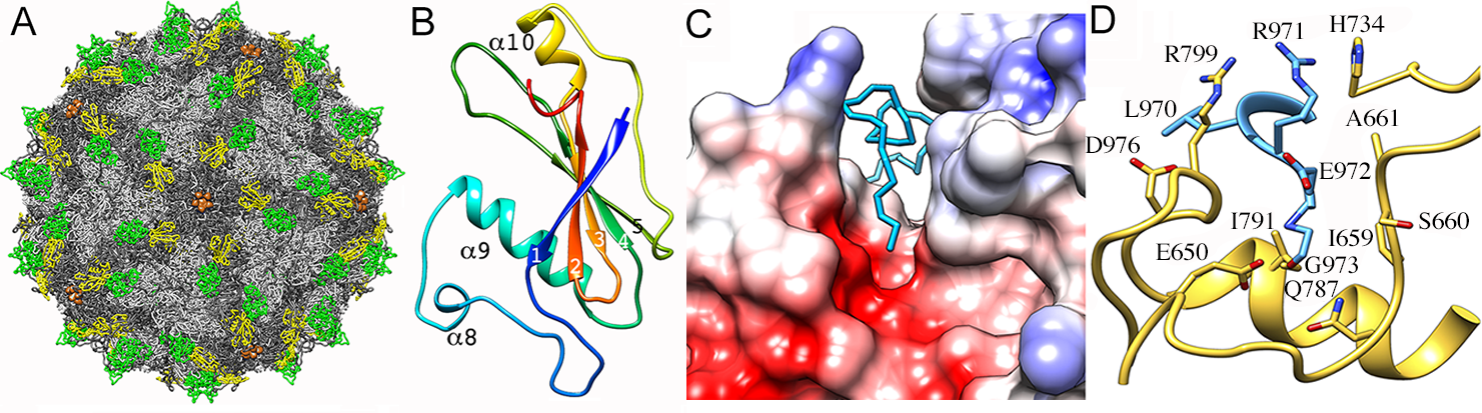
Putative enzyme activity on the RnQV1 outer capsid surface. (A) The P2 Asp227-Tyr350 domain (yellow) and P4 Val648-His805 domain (green) of a heterodimer are shown on the outer RnQV1 surface. P4 plugs at the five-fold axes are shown (orange). (B) The P2 Asp227-Tyr350 domain, rainbow-colored, with the topology found on the capsid surface. The five anti-parallel β-strands are indicated (1–5), as are the three helices (α8, α9, α10). (C) The P4 surface cleft (shown with electrostatic potentials) in which the P2 C terminus is located (blue, sticks). (D) Close-up of the P2 C-terminal segment (blue) in the P4 surface cavity (yellow), oriented as in c. Residues are displayed as sticks.

The conserved fold in P2 and P4 had two internal peptide insertion sites facing the outer capsid surface. For P2, these segments are Ser204-Asp365 (162 residues) in insertion site 1 and Ser599-Asp849 (251 residues) in insertion site 2 and for P4, the 89-residue His239-Tyr327 and the 367-residue Gly563-Thr929 segments, respectively (Fig 5A). Both insertion sites coincided with ScV-L-A Gag insertion sites, and one with the single-insertion site of the PcV CP domains (S4 and S5 Figs).

One interesting possibility is that the P2 and P4 insertions have associated enzymatic activities. The P2 Asp227-Tyr350 domain (in insertion site 1) is a twisted β-sheet of five antiparallel β-strands and three α-helices (Fig 5B). Analyses with Fatcat, Cath and Dali servers showed that this P2 domain has structural similarity, in addition to ferredoxin, to gelsolin and profilin (S3 Movie), two actin-binding proteins with a variety of actin regulatory functions [30]. The equivalent P4 segment is rather small, an 89-residue segment that includes the α-helices α7-α8-α9.

The P4 Val648-His805 domain (in insertion site 2) has a surface cavity in which the last visible P2 C-terminal residue, Gly973, contacts P4 Ile791 (Fig 5C and 5D). Preceding Gly973, the α29 helix and a 43-residue loop are positioned in a defined area on the P4 outer surface (S4 Movie). We hypothesize that this surface crevice in P4 participates in proteolytic processing of the P2 C-terminal domain.

### Protein-protein interactions that stabilize the RnQV1 capsid

**Fig 6:**
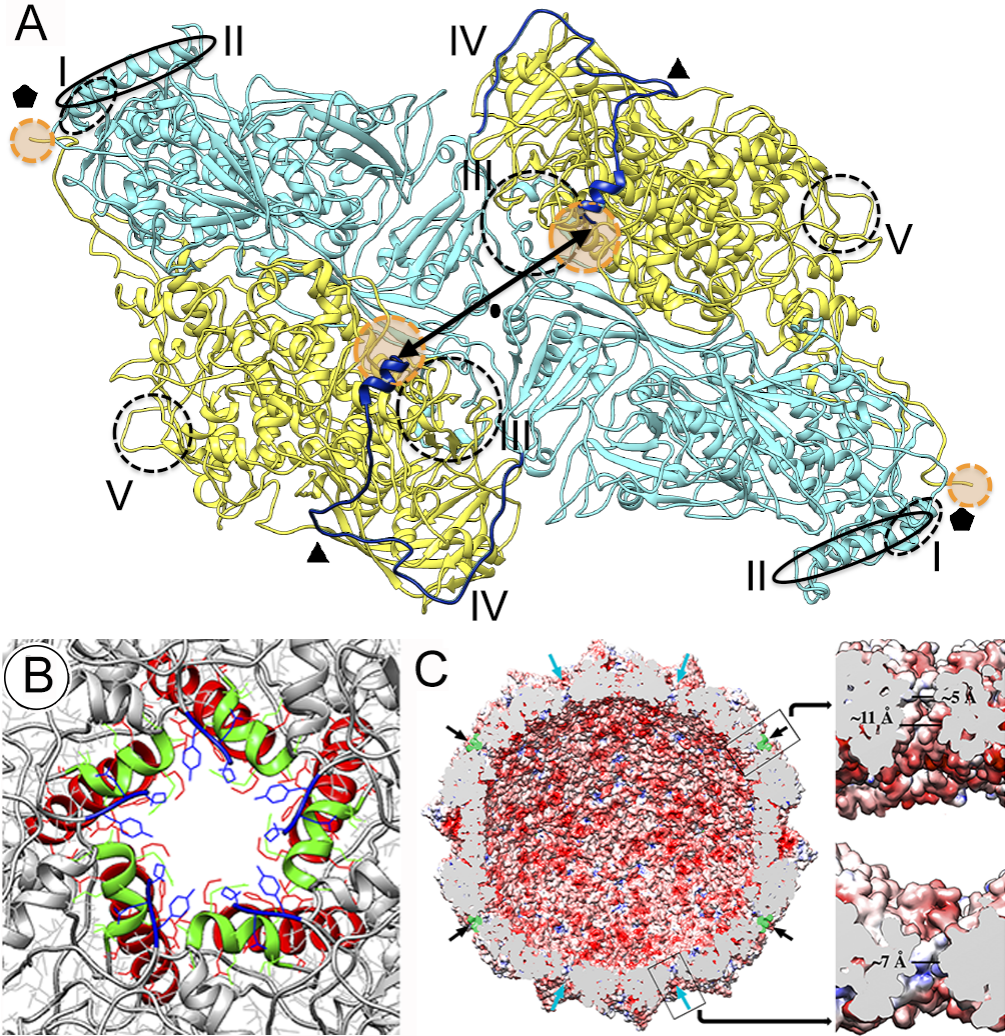
Molecular interaction and pore structure in the RnQV1 capsid. (A) Intra- and intersubunit interactions between P2 (blue) and P4 (yellow) (top view). Two P2-P4 heterodimers related by a two-fold symmetry axis (oval) are shown. Type I-V molecular hooks are indicated; type II (continuous oval) and IV hooks (dark blue line) are located on the outer capsid surface, hooks I (dashed oval), III and V (dashed circles) on the capsid inner surface. Small orange circles near the five-fold axis indicate the flexible, invisible C-terminal region of P4; large orange circles near the two-fold axis indicate the cleaved C-terminal region of P2, whose last visible residues are separated by 45 Å (double arrow). (B) Pores at the five-fold axes (top view). P2 α2 (red) and α5 (green) helices and P4 Lys1005 (blue) are indicated. Residues that define the pore opening are modeled as stick; P2 Lys27 (red) and Arg150 (green) and P4 Tyr1003 and Lys1005 (blue). (C) RnQV1 capsid inner surface represented with electrostatic potential, showing negative (red) and positive (blue) charge distribution. Arrows indicate capsid pores at the five-fold (black) and three-fold (blue) axes. Plugs of density of P4 flexible regions (green) are shown occluding the five-fold pores. Boxes, magnified views of the five- (top) and three-fold (bottom) pores showing charge distribution on channel walls (slabbed side view). Opening sizes are indicated.

Whereas contact interface between P2 and P4 has a surface of 7742 Å^2^, the contact surface area of each P2-P4 heterodimer with the surrounding heterodimers is 15,989 Å^2^. In addition to the numerous weak interactions in the buried intra- and interdimeric surfaces that stabilize the capsid, there are five molecular hooks formed by short loops or α-helices that extend further on the rough contact surfaces, and contribute to pentamer stability (Fig 6A, labeled I-V). Hook I, mediated by the P2 α2 helix (Gly11- Lys27), is a molecular swap between related P2 five-fold subunits. The α2 helices also form the broad opening of the channel on the capsid inner surface (Fig 6B, red helices). Another interdimeric hook, in this case between P2 and P4, is formed by P2 helix α6 (Thr154-Arg17) on the outer surface (hook II). Within the P2-P4 heterodimer, the P2 Leu883-Cys904 segment (which includes two short α-helices, α27 and α28) and its C-terminal end (Gly913-Gly973 segment) embrace the P4 inner and outer surfaces, respectively (Fig 6A, hooks III and IV). Hook V interdimeric connections are P4-mediated, in which loop Gln963-Gly976 interacts with the P2 inner surface.

These numerous reinforcements indicate that once dimers are formed, the pentamer constitutes the most stable capsid assembly unit. P2 C-terminal ends are relatively close to each other at the two-fold axes, separated by 45 Å (Fig 6A, double arrow). The missing 383-residue P2 C-terminal segment (Fig 6A, large orange circles) might constitute an external scaffolding domain.

### Pores through the RnQV1 capsid

Pores are located at the five- and three-fold axes (Fig 6C). The electrostatic potential of the inner capsid surface showed a very negatively charged surface (Fig 6C) that maintains RNA density ~25 Å from the capsid surface [25]. Except for the Penicillium chrysogenum virus (PcV) capsid (with positively charged regions in the inner surface that maintain RNA density in close contact), this feature resembles that of other single-shelled T = 1 capsids of dsRNA mycoviruses, as well as reo- and picobirnavirus (S6 Fig). The relatively low packed genome density in RnQV1 [25] and other fungal dsRNA viruses [16, 19, 31] could help minimize electrostatic repulsion between dsRNA and the acidic inner capsid surface.

The P2 α2 helix forms the inner opening of the pores (~11 Å-diameter hole) at the five-fold axis (Fig 6B, red helices; Fig 6C, black arrows); these openings narrow to ~5 Å diameter between the Lys27 side chains (Fig 6B, red). The P2 α5 helices, which are tangential to the capsid surface (Fig 6B, green helices), form the channel wall, maintaining the ~5 Å-diameter hole between Arg150 side chains (Fig 6B, green). The last visible residue of the P4 C termini, Lys1005, is surface-exposed and ends at the external pore opening (Fig 6B, blue). The P4 54-residue C-terminal segment, not traceable from the cryo-EM density map, is partially ordered near this surface opening, and acts as a molecular plug (Fig 6C, green; S7 Fig). Pores at the three-fold axis left a ~7 Å-diameter hole (Fig 6C, blue arrows, bottom right). These structural features indicate that exit of ssRNA viral transcripts (as in other dsRNA viruses) would require conformational changes in P2 and/or P4 segments.

### Structural comparison of P2 and P4 with other dsRNA mycovirus capsid proteins

The structural features described thus for the ~300-residue conserved region between P2 and P4 (P2* and P4*) indicate that it could have evolved from the ancestral domain of the dsRNA virus lineage. We compared P2* and P4* with the CP of several mycoviruses such as L-A virus of the yeast *Saccharomyces cerevisiae* (ScV-L-A), a totivirus with a single genome dsRNA segment, and PcV, a chrysovirus with four monocistronic dsRNA segments. These mycoviruses, including RnQV1, are transmitted intracellularly and have no outer shell on the rough outer surface of their T = 1 capsids, in contrast with the smooth outer surface of reo- and cystovirus capsids.

**Fig 7:**
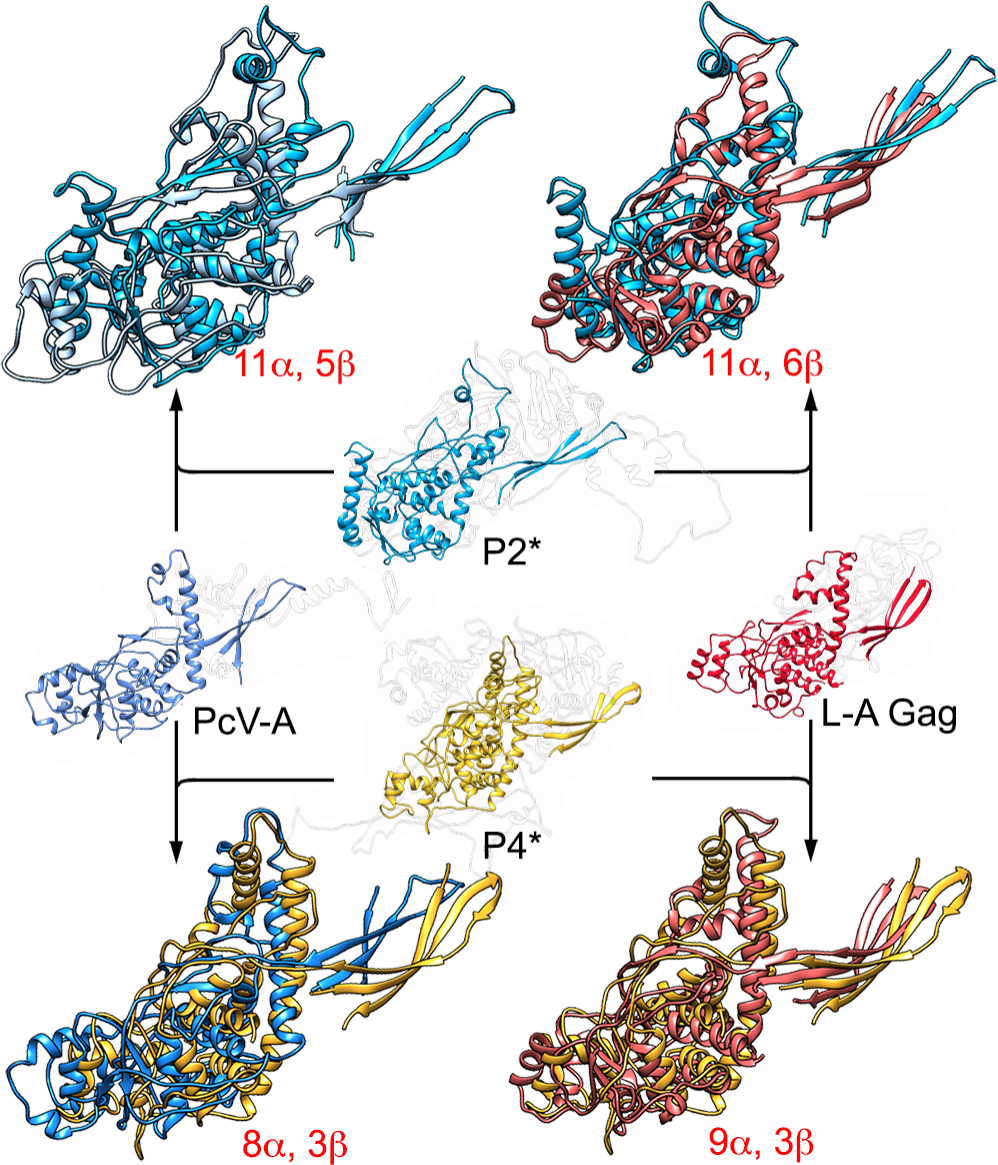
Structural homology of P2 and P4 with PcV CP A domain and L-A virus Gag CP. P2* and P4* (center) were structurally aligned with PcV CP A domain (PcV-A, left) and L-A virus Gag (L-A Gag, right). Center structures indicate conserved SSE within the dsRNA viruses or BTV-like lineage. P2* is aligned with PcV-A (blue and light blue, top left) and with L-A Gag (blue and pink, top right). P4 is aligned with PcV-A (yellow and blue, bottom left) and P4 with L-A Gag (yellow and pink). Total numbers of SSE with close relative spatial locations are indicated.

Dali structural alignments between the Gag CP of ScV-L-A or the PcV A domain with P2* indicated notable structural similarity (Fig 7, Table 1, S4 Fig). When P2* and Gag or the A domain were superimposed, 11 α-helices and 5–6 β-strands showed close relative spatial positions and required only minor local adjustments for overlap (S5 and S6 Movies). Comparable analysis of Gag and the A domain with P4* showed superimposition of 8–9 α-helices and 3 β-strands (Fig 7, Table 1, S5 Fig, S5 and S6 Movies). We performed similar analyses between PcV B domain and P2* and P4* (Table 1). The most conserved α-helices between P2* and P4* with Gag and A and B domains were located near the five-fold axis. The P2* and P4* β-sheet structure, formed by three relatively long β-strands and found respectively at the two- and three-fold axes, was also very conserved in all mycoviruses tested. The P2 SIID (residues 738–848) and P4 (817–929) were detected neither in CP of the other mycoviruses nor of higher dsRNA viruses.

## Discussion

The 120-subunit T = 1 capsid is an almost ubiquitous architecture of the protein layer that surrounds the dsRNA virus genome. The asymmetric unit is a CP homodimer with a conserved fold exemplified by the BTV CP fold. In addition to capsid assembly constraints, this fold is necessary for capsid function, to organize the viral genome and replicative machinery, and act as a molecular sieve to evade intracellular host defense mechanisms.

Here we show the RnQV1 capsid at near-atomic resolution; its asymmetric unit is a dimer of two distinct proteins, P2 and P4 (2415 amino acid residues total) and is thus the most complex T = 1 capsid known so far. Despite their lack of sequence similarity, structural alignment of P2 and P4 showed two common domains in the shell region, SID and SIID. Whereas SID is preserved in other fungal dsRNA viruses (toti- and chrysoviruses) [32] as well as in reoviruses, SIID is unique to P2 and P4. P2 and P4 SID (P2* and P4*) have 11 α-helices (including a long α-helix tangential to the capsid surface) and a β-sheet with similar tertiary structures. These SSE show the folding signature of most dsRNA virus CP. SIID, which has 3 α-helices and a short β-sheet, is much smaller than SID (~110 vs. ~350 residues). Whereas P2* and P4* preserved folds are compatible with a common CP ancestor of dsRNA viruses, P2 and P4 SIID suggest a common origin for P2 and P4.

Duplication of an ancestral gene for a CP with the BTV-like fold might have resulted in two separate (as in quadriviruses) or covalently joined folds (as in chrysoviruses). This event could direct assembly of a T = 1 capsid with 120 subunits or domains with a dimer as the asymmetric unit, a necessary arrangement for dsRNA replication/transcription. Separate and joined folds are found in other virus families, which implies a recurrent evolutionary event. Whereas adeno- and comovirus CP are the result of a joined fold [33, 34], the picornavirus capsid is assembled from three different proteins with very similar folds, although with negligible sequence similarity [4]. Once the 120-subunit capsid was well established, later divergent evolutionary events would have introduced additional changes in each copy, or even the complete removal of one of them, giving a CP that assembles as a dimer of unfused identical monomers (the so-called A and B subunits). Alternatively, the ancestral CP could have initially acquired dimer assembly ability, followed by gene duplication; domain swapping could be another mechanism by which dimers and oligomers assemble from monomers [35].

Besides the subunit interactions in the solvent-buried surfaces, there are five molecular hooks in the RnQV1 capsid that probably stabilize assembly of five dimers into a pentamer, which has major effects on the RnQV1 assembly pathway. In reo-, toti-, and chrysoviruses, all built from asymmetric dimers, CP are arranged approximately as parallel dimers [8]; these viruses probably initiate capsid assembly from pentamers of dimers. Partiti- and picobirnavirus capsids (built from quasi-symmetric dimers), as well as the cystovirus capsid, are thought to use dimers of CP dimers as assembly intermediates [10, 16]. The P2 and P4 asymmetric dimer lacks the 383-residue P2 C terminus. This cleaved polypeptide fragment is predicted to be helical; we hypothesize that it acts as an external scaffold domain covalently bound to P2. Given that the last P2 C-terminal residue is only 45 Å from a neighboring P2 C terminus end, dimerization of these scaffolds could direct pentamer accretion or, more probably, assembly of two dimers into a tetramer (S8 Fig). The immature RnQV1 capsid would thus have 30 dimeric α-helical densities extruding radially relative to the capsid shell. Generation of cDNA clones for P2 and P4 might allow introduction of mutations in the P2 processing site to assemble immature virus-like particles.

Dimerization and high α-helical content are ubiquitous features of scaffold proteins [36, 37]. External scaffold proteins are reported for capsids of bacteriophages P4 [38, 39] and ϕX174 [40]. The protease-free Prohead-1 map of bacteriophage HK97 shows rod-shaped densities intruding on its internal surface, which correspond to the scaffold domains fused to the CP N termini [41]. The P1 CP of bacteriophage ϕ8, a cystovirus, is found as a soluble tetramer in an *in vitro* assembly system [42].

In multilayer capsids of dsRNA viruses (as in Reo- and Cystoviridae), the T = 1 capsid has a smooth outer surface that serves as nucleation site for the T = 13 surrounding capsid. Fungal dsRNA viruses are transmitted by cytoplasmic interchange and commonly confined to the host, and have a single-shelled T = 1 capsid with an uneven outer surface. The original roles of the capsid, such as receptor binding and/or genome protection, have given way to new abilities reflected in altered capsid surface features. In RnQV1, in addition to the N and C termini, we detected two well-defined insertion sites into which insertions of ~90–370 residue segments lead to variations. These preferential insertion sites, which face the outer capsid surface as also seen in PcV and ScV-L-A, are conserved among dsRNA viruses and are probably ancient.

The 41-residue insertion in ScV-L-A Gag insertion site 1 is responsible for the decapping that transfers cap structures from the 5’ end of cellular mRNA to the 5’ end of viral RNA [43, 44]; it coincides with the 162-residue insertion in P2, which has structural similarity to proteins involved in actin cystoskeleton regulation. In P4 insertion site 2, the 367-residue insertion has a surface cleft with putative proteolytic activity, which our structural results suggest is primarily exerted on the P2 C terminus, although other proteins might be targeted. PcV insertions are much smaller and no activity or structural similarity has yet been found. The similarity of 3D locations of these inserted peptides/domains could indicate a mechanism for acquisition of new functions without altering the basic structural core of the dsRNA virus CP.

The CP of many tailed dsDNA phages with the canonical HK97-like core (also termed Johnson fold) has additional domains or defined regions with specific functions related to capsomer or capsid stability (reviewed in [45]). The human cytomegalovirus (HCMV), a herpesvirus, has a 1370-residue CP folded into seven domains [46] and thus shares some similarity with the RnQV1 CP. The HCMV Johnson fold or floor domain at the shell has a six-domain protruding tower that interacts with several outer CP and has specific functions based on the virus transmission cycle. The Johnson fold has a five-stranded β-core that acts as an organizational hub of the major CP; the additional domains are considered modular insertions into the peripheral loops [46]. In this context, conserved α-helices and/or the β-sheet structure preserved in the dsRNA virus basic fold might constitute a similar functional center.

Analyses of the RnQV1 outer capsid surface showed that the insertional domains for these activities are proximally aligned and could form an efficient assembly line of molecular machines. To our knowledge, mycovirus capsids are not yet exploited for nanotechological applications; our structural studies might be a step toward future protein-based nanocages that incorporate active whole protein in a controlled platform.

## Materials and methods

### Virion preparation

RnQV1 virions were purified from Rosellinia necatrix strain W1118 by two cycles of differential centrifugation and one rate zonal centrifugation in a sucrose density gradient, as described [25, 47]. The two UV-absorbing bands, corresponding to empty (top) and to a mixture of empty and full particles (bottom), were collected separately by side puncture, diluted with buffer A (50 mM Tris-HCl buffer, pH 7.8, 5 mM EDTA, 150 mM NaCl), and concentrated by centrifugation (106,000 x g, 12 h, 4°C) [25]. Concentrated particles were loaded on a 36% CsCl cushion and ultracentrifuged (180,000 x g, 110 min, 4°C). The purified empty particles, derived from a mixture of full and empty particles, were homogeneous and appropriate for further cryo-EM analysis.

### Cryo-EM

Purified RnQV1-W1118 empty particles (5 μl) were applied to R2/2 300 mesh copper-rhodium grids (Quantifoil Micro Tools, Germany) and vitrified using a Leica EM CPC cryofixation unit. Data were collected on a FEI Titan Krios electron microscope operating at 300 kV and images recorded on a FEI Falcon II detector at a calibrated magnification of 104,478, yielding a pixel size of 1.34 Å. A dose rate of 28 electrons/Å^2^/s and 1.4 s exposure time were used to record 1,125 24-frame movies with a defocus range of 0.7 to 3.5 μm.

### Image processing

To correct for beam-induced movement, the 18 central frames of each movie were aligned using whole-image motion correction [48], after which local movements were corrected using an Optical Flow approach [49]. Contrast transfer function parameters of each averaged movie were determined using CTFFIND3 [50]. Images showing astigmatism and/or motion signs were discarded, maintaining a total of 996. General image processing operations were performed using Xmipp [51] [http://xmipp.cnb.csic.es/] and Relion [52] [http://www2.mrc-lmb.cam.ac.uk/relion/index.php/Main_Page]. Graphics were produced by UCSF Chimera [53] [http://www.cgl.ucsf.edu/chimera/]. A total of 53,683 particles were picked with the Xmipp automatic picking routine and 37,531 particles were selected manually. Alternatively, Relion reference-free 2D classification was used to discard bad particles and a similar data set of 42,267 particles was obtained. 3D classification with Relion resulted in four classes using the RnQV1-W1075 structure [25] (EMD-3437) and low-pass filtered to 40 Å as initial reference. Further final Relion iterative auto-refine was performed including 19,194 particles (for the best 3D class), 28,101 (including the two best 3D classes), or including all four 3D classes. The Relion particle polishing protocol was used for per-particle motion correction by fitting linear tracks and to weight each frame with a different B-factor based on a dose-dependent model for radiation damage. The best resolution, 3.70 Å based on the gold-standard FSC = 0.143 criterion corrected for the effects of a soft mask on the FSC curve using high-resolution noise substitution, was obtained using the complete data set of 3D-selected particles [54]. The final density map was corrected for the modulation transfer function (MTF) of the detector and sharpened by applying the estimated B-factor [55]. Local resolution variations were calculated using ResMap [56].

### Atomic model building and model refinement

The backbone of each polypeptide chain in a single asymmetric unit of the 3.7 Å cryo-EM map was built *de novo* using Coot crystallographic modeling [57]. A poly-Ala sequence was entered manually for each protein and clear densities of bulky side chains were marked. The comparison of predicted and observed SSE was used as starting point to register the amino acid sequence for P2 and P4 proteins. In this process, we used the P4 region of the helices α14-α16 (residues 472–541), which matched the four predicted helices α15-α 18 (residues 476–548), with focus on the bulky side chains at residues 522–526. Once the sequence was registered, positions of the main and side chains were adjusted manually and fit of the atomic model to the density map was improved by iterative cycles of model rebuilding using Coot [57]. To evaluate and improve model accuracy, we used Refmac5 [58] to eliminate clashes and inconsistencies in geometry. Comparison of coordinates before and after Refmac5 processing was used to locate regions that required further refinement. In addition, the geometry of the model was tested iteratively with Coot validation tools, which helped to detect and correct residues outside allowed regions in the Ramachandran plot. The final modeled coordinates were refined in 10 additional cycles of Refmac5, and Coot. The Ramachandran plot showed 2.59% of residues in disallowed regions.

### Model analysis

The electrostatic potential for the RnQV1-W1118 capsid was calculated using DelPhi software [59] and surface-colored with UCFS Chimera. The Dali server [60] (http://ekhidna.biocenter.helsinki.fi/dali_server/start) was used for structural alignment between RnQV1 CP P2 and P4 proteins and their overlap with PcV CP A and B domains as well as L-A Gag CP. We used FATCAT [61] (http://fatcat.burnham.org), CATH [62] (http://www.cathdb.info) and Dali servers to search for structures related to the P2 Asp227-Tyr350 domain.

### Protein identification by MALDI-TOF/TOF mass spectrometry (MS)

Protein gel bands from sucrose gradient fractions enriched with RnQV1 capsids were excised from Coomassie-blue stained gels, placed in a 96-well plate and digested using the DP Proteineer digestion robot (Bruker Daltonics) with trypsin (5 h, 37°C) as described [63]. Resulting peptides were analyzed by MALDI-TOF/TOF using an ABI Sciex 4800 Proteomics Analyzer. To submit the combined peptide mass fingerprint (PMF) and MS/MS data to MASCOT software v.2.5.1 (Matrix Science, UK), GPS Explorer v4.9 was used, searching the nonredundant NCBI protein database (NCBInr_20160610).

### Data deposition

The RnQV1-W1118 cryo-EM map is deposited in the Electron Microscopy Data Bank (accession no. emd-3619) and the atomic coordinates of the RnQV1-W1118 P2 and P4 proteins are deposited in the PDB (ID code 5ND1).

## Supporting information

S1 Fig Resolution of the RnQV1capsid and quality of the RnQV1 P2 and P4 density maps. (A) Surface-shaded RnQV1 capsid (left) and central slab of density (right) viewed along a twofold axis. The surface is colored based on local resolution as indicated in the color key (values in Å). (B) Cryo-EM density map of the RnQV1 capsid asymmetric unit. Backbones of P2 (yellow) and P4 (blue) are superimposed on the density map. (C, D) Regions of the cryo-EM density map (grey mesh), with atomic models of (C) helices P4 α25 (yellow), P2 α6 (blue) and (D) two β-sheets of P4 (top, with β-strands 1, 2 and 9) and P2 (bottom, with β-strands 1, 2 and 20). Atomic models are shown as ribbons and sticks with amino acid residues labeled.
(TIF)

S2 Fig Mass spectra of P2 and P4. Positive ion mode mass spectra of RnQV1 (A) P2 and (B) P4 bands analyzed by MALDI ionization. Mass spectra showed peaks in the mass/charge (m/z) 900–3500 range.
(TIF)

S3 Fig Structural similarity of P2 and P4 SIID. Top, ribbon diagrams of P2 (left) and P4 (right) SIID, rainbow-colored from blue (N terminus) to red (C terminus). Bottom, sequence alignment of P2 Cys738-Ser848 (blue) and P4 Gly817-Thr929 (yellow) segments. α-helices (rectangles) and β-strands (arrows) are rainbow-colored from blue (N terminus) to red (C terminus) for each SIID.
(TIF)

S4 Fig Structural alignment of P2 with PcV domain A and L-A virus Gag CP. (A) Dali sequence alignment of P2 (blue) and PcV domain A (orange). The α-helices (rectangles) and β-strands (arrows) are rainbow-colored from blue (N terminus) to red (C terminus). Triangles indicate nonaligned segments (sizes indicated). (B) Dali sequence alignment of P2 (blue) and L-A virus Gag (red). Triangles indicate nonaligned segments (sizes indicated).
(TIF)

S5 Fig Structural alignment of P4 with PcV domain A and L-A virus Gag CP. (A, B) Dali sequence alignments of P4 (yellow) and PcV domain A (orange) (A), and P4 (yellow) and L-A virus Gag (red) (B). The α-helices (rectangles) and β-strands (arrows) are rainbow-colored from blue (N terminus) to red (C terminus). Triangles indicate nonaligned segments (sizes indicated).
(TIF)

S6 Fig dsRNA virus T = 1 inner capsid surfaces with electrostatic potentials. T = 1 capsids of Penicillium chrysogenum virus (PcV), L-A virus (L-A), Penicillium stoloniferum virus F (PsV-F), picobirnavirus (PBV), Rosellinia necatrix quadrivirus 1 (RnQV1), rotavirus, bluetongue virus (BTV), cytoplasmic polyhedrosis virus (CPV), and orthoreovirus, viewed along a two-fold axis of icosahedral symmetry. The inner surface charge representations of this non-exhaustive group of dsRNA virus T = 1 capsids show the distribution of negative (red) and positive (blue) charges. dsRNA densities and other packaged proteins (such as RNA polymerases) were removed computationally.
(TIF)

S7 Fig C-terminal P4 density occludes the five-fold axis channels of the RnQV1 capsid. (A) Central section of the RnQV1 capsid 3D reconstruction viewed along a two-fold axis (protein is dark). Arrows indicate the five- (5), three- (3) and two-fold (2) icosahedral symmetry axes. (B) Magnified view of the box in A. Atomic model regions of P2 (blue) and P4 (yellow) are superimposed on the protein density (black) The last visible P4 residue (K1005) is indicated in two P4 molecules. (C) Five copies of P2 (blue) and five of P4 (yellow) are superimposed on the density map around a five-fold axis. The five P4 Lys1005 (red) are followed by five partially disorganized densities (yellow) that occlude the five-fold axis channel, in which it was not possible to include additional P4 residues.
(TIF)

S8 Fig Proposed assembly intermediates for 120-subunit immature RnQV1 capsid. (A) Immature RnQV1 capsid. The 30 dimeric rod-shaped densities (orange) represent the processed P2 C-terminal α-helical domains. Capsid shell colored as in Fig 5. (B) A non-processed P2-P4 heterodimer. The C-terminal regions (orange) are shown as a rod-like density (right) for P2 and a spherical density (left) for P4. (C) A non-processed tetramer as the basic assembly unit. The P2 C-terminal α-helical domain contacts an equivalent region of a neighboring dimer, which might promote this type assembly. (D) A non-processed pentamer as the basic assembly unit. Wavy lines indicate that the P2 C-terminal α-helical domain is flexible and unstable.
(TIF)

S1 Table Analysis by MALDI-TOF/TOF MS and MS/MS of P2 and P4 proteins. (DOCX)

S1 Movie Structural alignment of RnQV1 capsid proteins P2 and P4, showing superimposed α-helices and β-strands, rainbow-colored from blue (N terminus) to red (C terminus) for each protein (insertions or non-superimposed regions, white). (MP4)

S2 Movie Structural alignment of P2 and P4 SIID, showing superimposed α-helices and β-strands, rainbow-colored from blue (N terminus) to red (C terminus) for each domain. (MP4)

S3 Movie Structural alignment of P2 Asp227-Tyr350 domain with gelsolin, profilin, and the ferredoxin-like domain, showing superimposable β-sheet and α-helices. (MP4)

S4 Movie The P2 C-terminal segment is located on the P4 outer surface, and the last C-terminus residue (Gly973) ends in a P4 surface crevice. P2 is shown as blue sticks and P4 surfaces are represented with electrostatic potentials, showing the distribution of negative (red) and positive (blue) charges.
(MP4)

S5 Movie Structural alignment of P2 (left) and P4 (right) with PcV domain A (center), showing superimposable α-helices and β-strands, rainbow-colored from blue (N terminus) to red (C terminus). (MP4)

S6 Movie Structural alignment of P2 (left) and P4 (right) with L-A virus Gag CP (center), showing superimposable α-helices and β-strands, rainbow-colored from blue (N terminus) to red (C terminus). (MP4)
