# Supplementary figures and images for: Acquisition of functions on the outer capsid surface during evolution of double-stranded RNA fungal viruses
Source: PLoS Pathog. 2017 Dec 8;13(12):e1006755. doi: 10.1371/journal.ppat.1006755 (PMC5738138; doi:10.1371/journal.ppat.1006755)

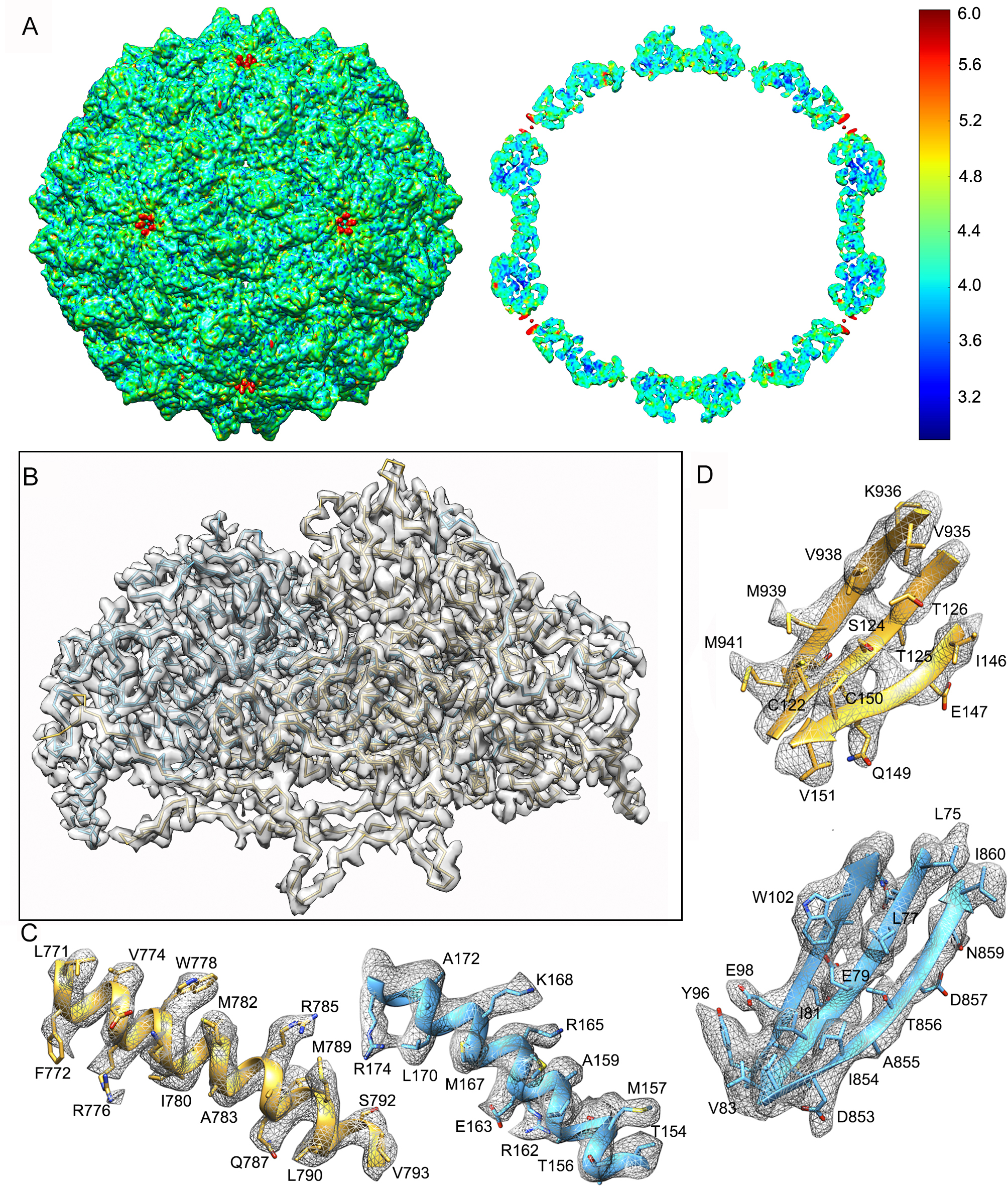

Supplement: S1 Fig — (A) Surface-shaded RnQV1 capsid (left) and central slab of density (right) viewed along a twofold axis. The surface is colored based on local resolution as indicated in the color key (values in Å). (B) Cryo-EM density map of the RnQV1 capsid asymmetric unit. Backbones of P2 (yellow) and P4 (blue) are superimposed on the density map. (C, D) Regions of the cryo-EM density map (grey mesh), with atomic models of (C) helices P4 α25 (yellow), P2 α6 (blue) and (D) two β-sheets of P4 (top, with β-strands 1, 2 and 9) and P2 (bottom, with β-strands 1, 2 and 20). Atomic models are shown as ribbons and sticks with amino acid residues labeled. (TIF) [file ppat.1006755.s001.tif]

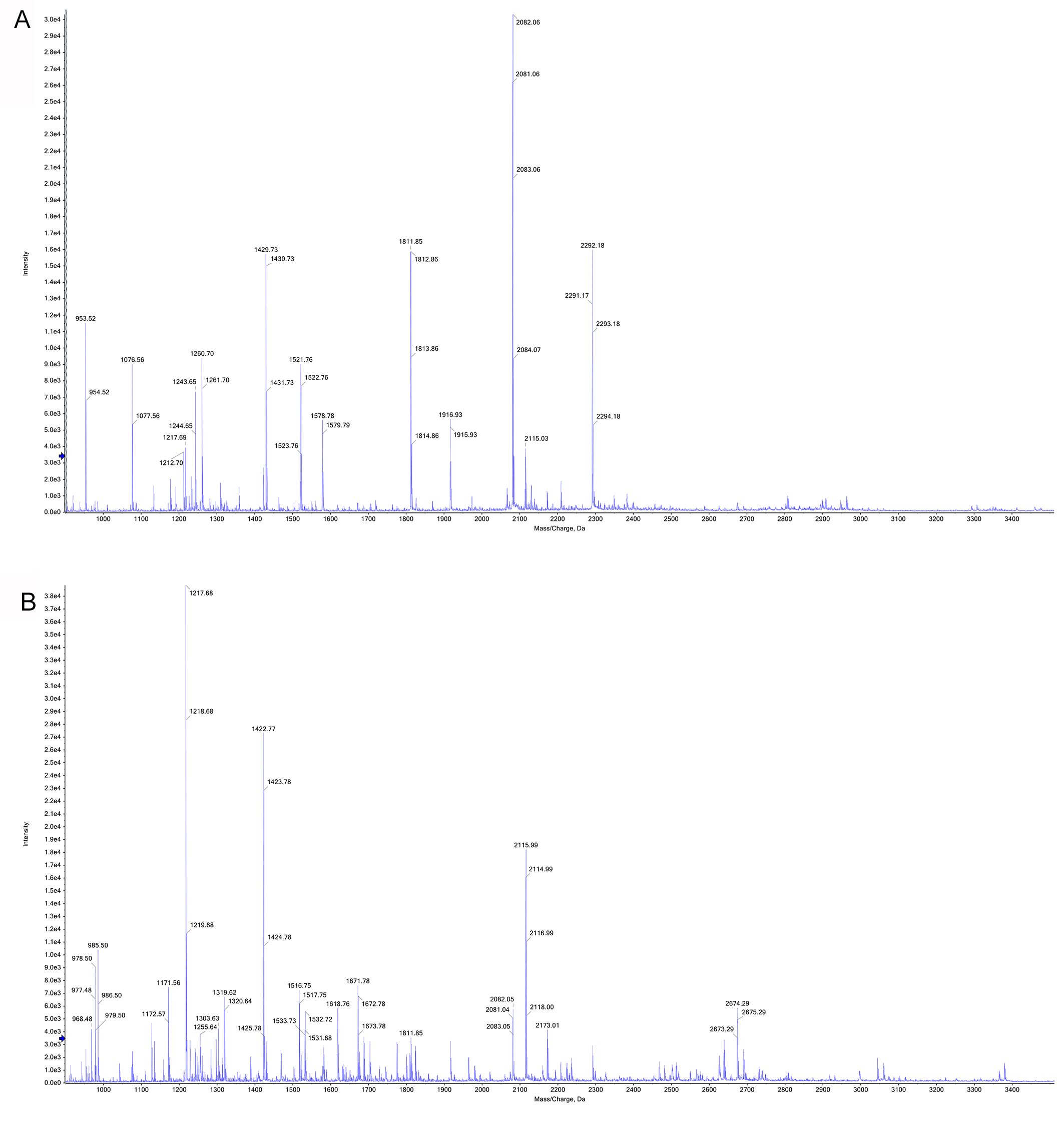

Supplement: S2 Fig — Positive ion mode mass spectra of RnQV1 (A) P2 and (B) P4 bands analyzed by MALDI ionization. Mass spectra showed peaks in the mass/charge (m/z) 900–3500 range. (TIF) [file ppat.1006755.s002.tif]

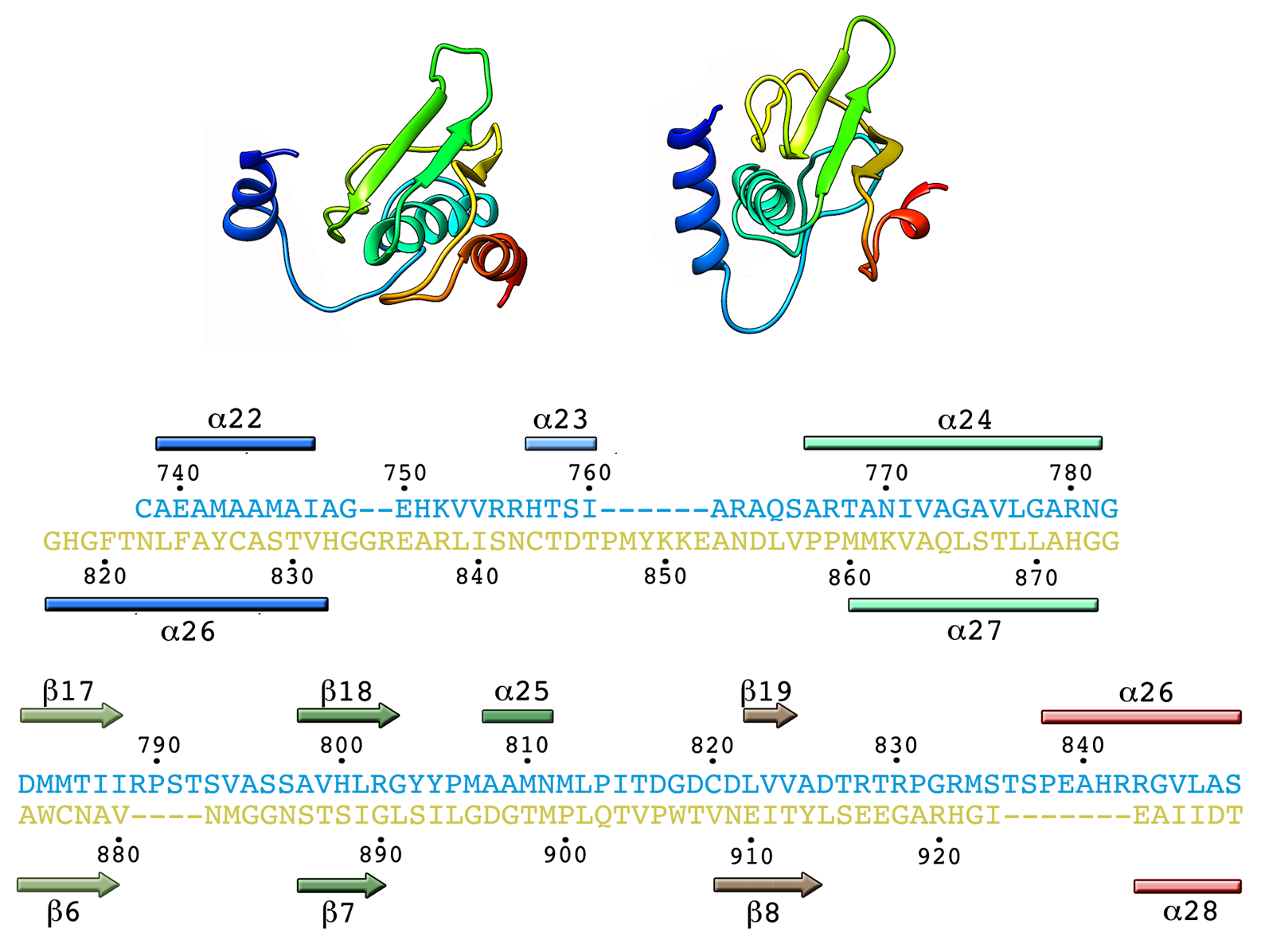

Supplement: S3 Fig — Top, ribbon diagrams of P2 (left) and P4 (right) SIID, rainbow-colored from blue (N terminus) to red (C terminus). Bottom, sequence alignment of P2 Cys738-Ser848 (blue) and P4 Gly817-Thr929 (yellow) segments. α-helices (rectangles) and β-strands (arrows) are rainbow-colored from blue (N terminus) to red (C terminus) for each SIID. (TIF) [file ppat.1006755.s003.tif]

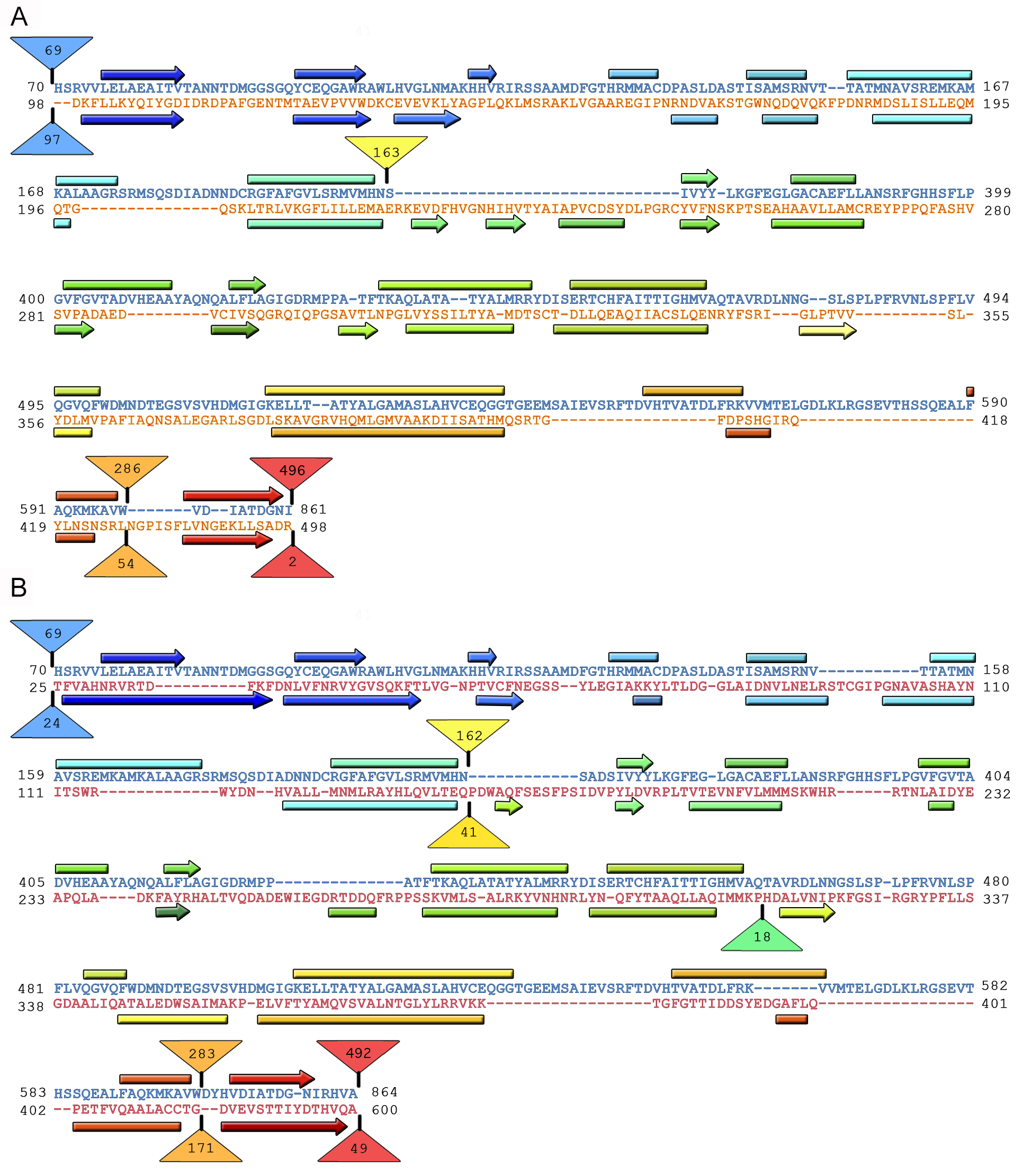

Supplement: S4 Fig — (A) Dali sequence alignment of P2 (blue) and PcV domain A (orange). The α-helices (rectangles) and β-strands (arrows) are rainbow-colored from blue (N terminus) to red (C terminus). Triangles indicate nonaligned segments (sizes indicated). (B) Dali sequence alignment of P2 (blue) and L-A virus Gag (red). Triangles indicate nonaligned segments (sizes indicated). (TIF) [file ppat.1006755.s004.tif]

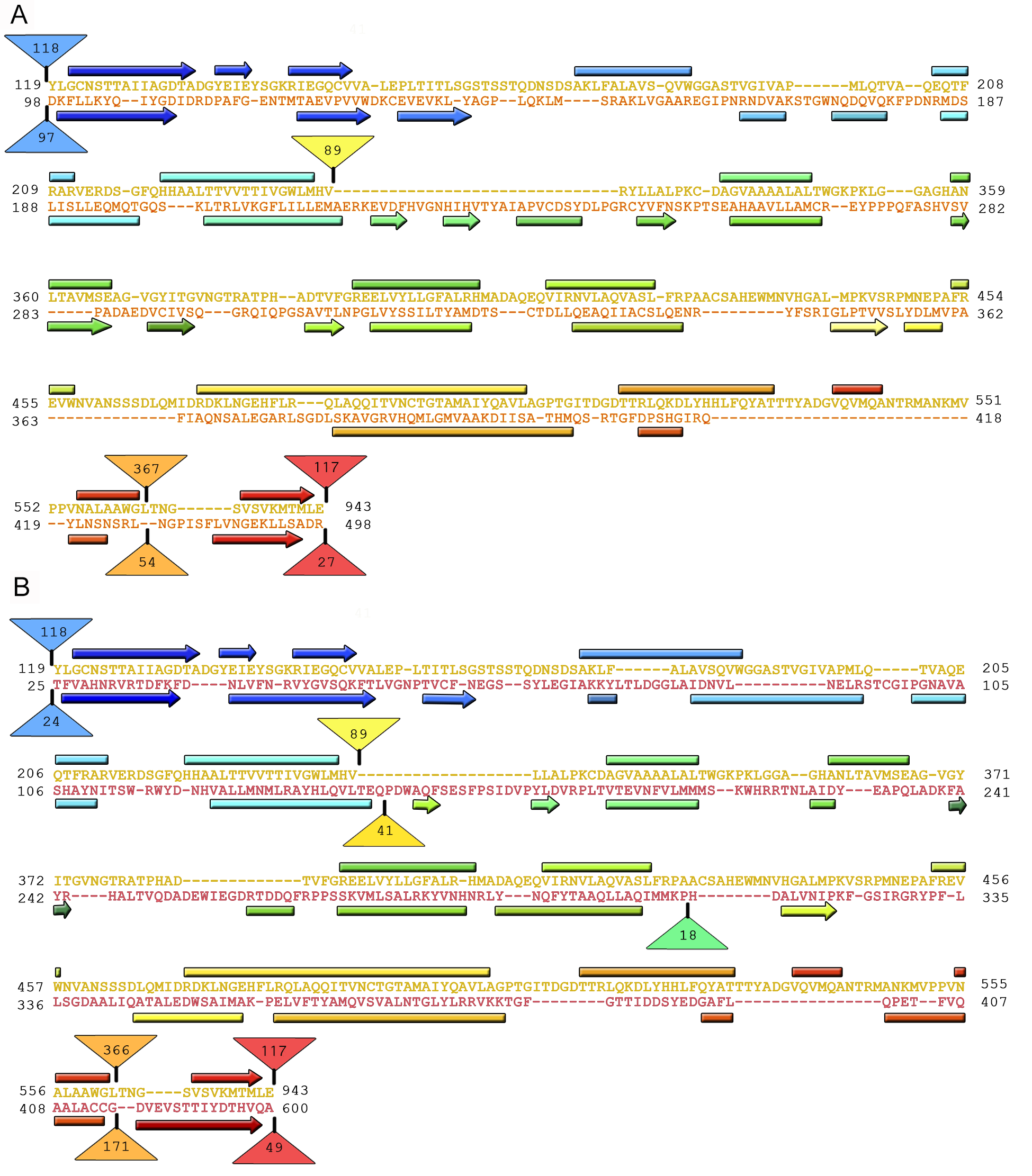

Supplement: S5 Fig — (A, B) Dali sequence alignments of P4 (yellow) and PcV domain A (orange) (A), and P4 (yellow) and L-A virus Gag (red) (B). The α-helices (rectangles) and β-strands (arrows) are rainbow-colored from blue (N terminus) to red (C terminus). Triangles indicate nonaligned segments (sizes indicated). (TIF) [file ppat.1006755.s005.tif]

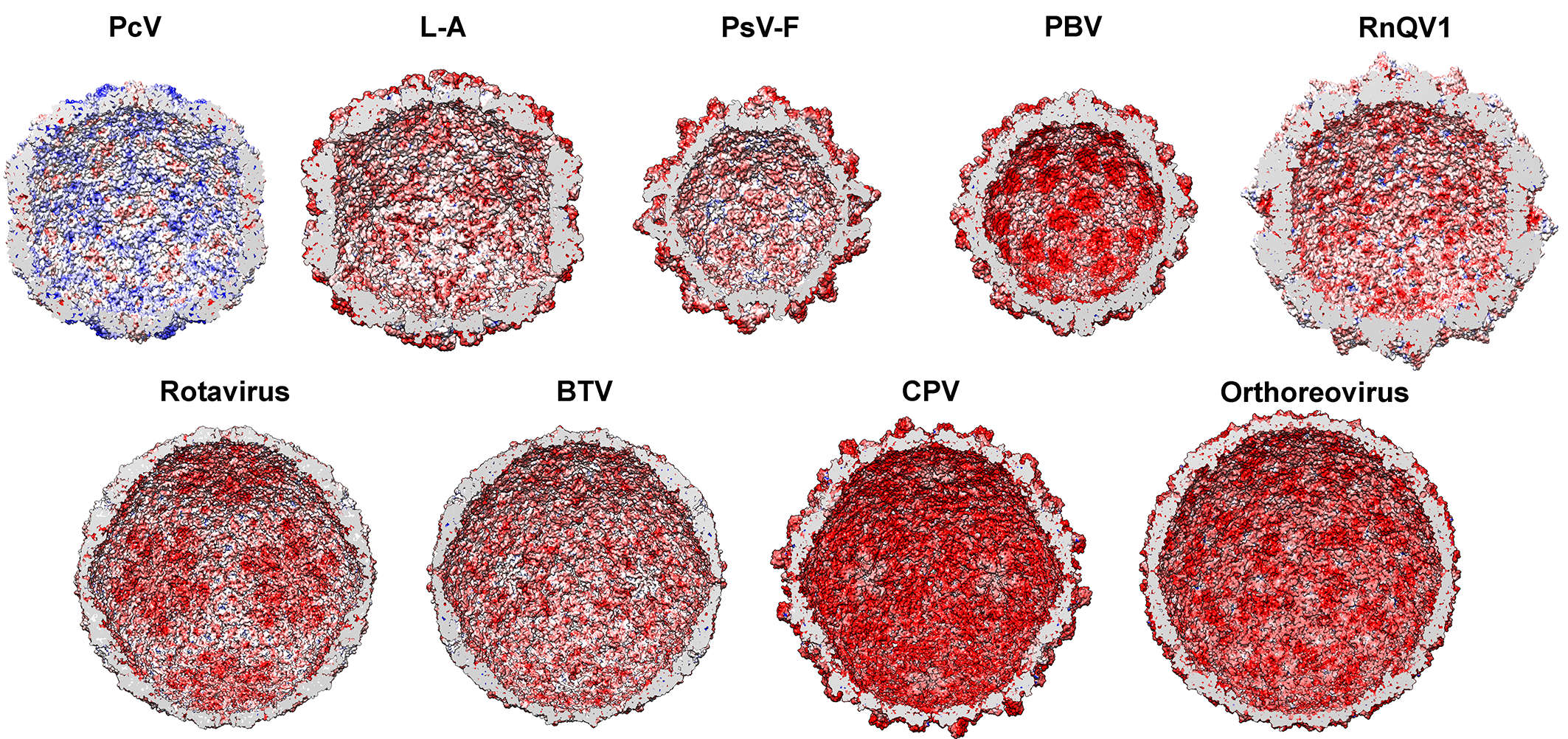

Supplement: S6 Fig — T = 1 capsids of Penicillium chrysogenum virus (PcV), L-A virus (L-A), Penicillium stoloniferum virus F (PsV-F), picobirnavirus (PBV), Rosellinia necatrix quadrivirus 1 (RnQV1), rotavirus, bluetongue virus (BTV), cytoplasmic polyhedrosis virus (CPV), and orthoreovirus, viewed along a two-fold axis of icosahedral symmetry. The inner surface charge representations of this non-exhaustive group of dsRNA virus T = 1 capsids show the distribution of negative (red) and positive (blue) charges. dsRNA densities and other packaged proteins (such as RNA polymerases) were removed computationally. (TIF) [file ppat.1006755.s006.tif]

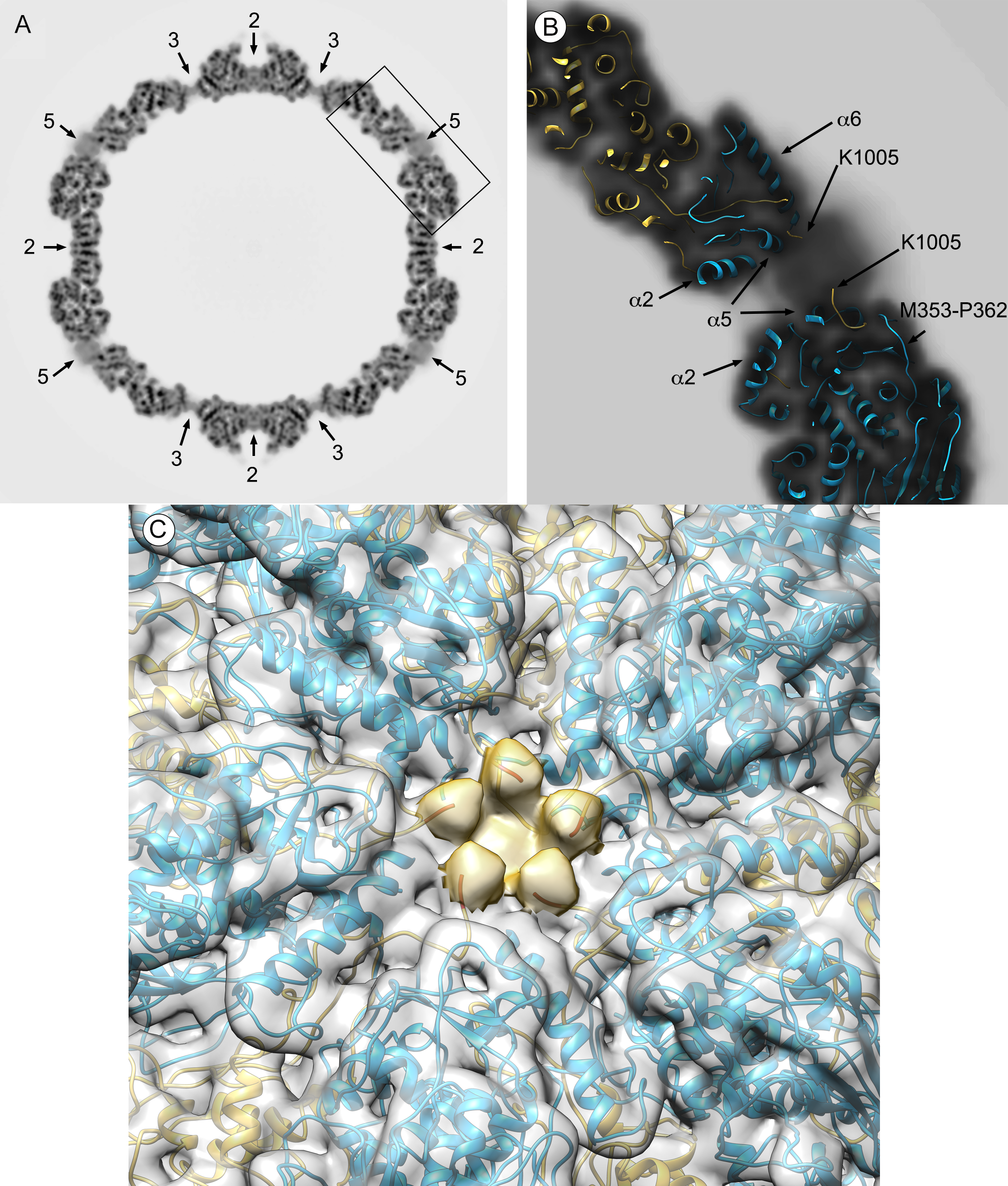

Supplement: S7 Fig — (A) Central section of the RnQV1 capsid 3D reconstruction viewed along a two-fold axis (protein is dark). Arrows indicate the five- (5), three- (3) and two-fold (2) icosahedral symmetry axes. (B) Magnified view of the box in A. Atomic model regions of P2 (blue) and P4 (yellow) are superimposed on the protein density (black) The last visible P4 residue (K1005) is indicated in two P4 molecules. (C) Five copies of P2 (blue) and five of P4 (yellow) are superimposed on the density map around a five-fold axis. The five P4 Lys1005 (red) are followed by five partially disorganized densities (yellow) that occlude the five-fold axis channel, in which it was not possible to include additional P4 residues. (TIF) [file ppat.1006755.s007.tif]

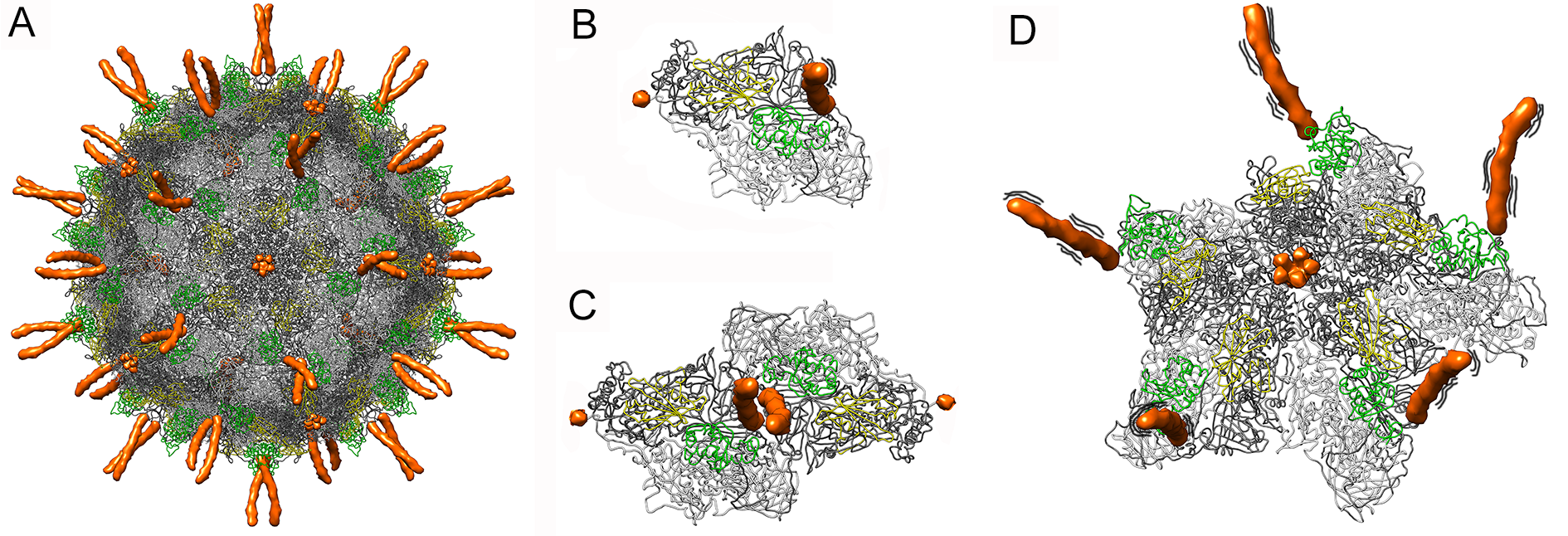

Supplement: S8 Fig — (A) Immature RnQV1 capsid. The 30 dimeric rod-shaped densities (orange) represent the processed P2 C-terminal α-helical domains. Capsid shell colored as in Fig 5. (B) A non-processed P2-P4 heterodimer. The C-terminal regions (orange) are shown as a rod-like density (right) for P2 and a spherical density (left) for P4. (C) A non-processed tetramer as the basic assembly unit. The P2 C-terminal α-helical domain contacts an equivalent region of a neighboring dimer, which might promote this type assembly. (D) A non-processed pentamer as the basic assembly unit. Wavy lines indicate that the P2 C-terminal α-helical domain is flexible and unstable. (TIF) [file ppat.1006755.s008.tif]
