# Supplementary material for: Acquisition of functions on the outer capsid surface during evolution of double-stranded RNA fungal viruses
Source: PLoS Pathog. 2017 Dec 8;13(12):e1006755. doi: 10.1371/journal.ppat.1006755 (PMC5738138; doi:10.1371/journal.ppat.1006755)
Supplement: S1 Table — (DOCX) [file ppat.1006755.s009.docx]

| Table S1 | | | | | | |
| --- | --- | --- | --- | --- | --- | --- |
| Protein Acc. | Protein | Matched Peptides MS(MS/MS) | Mascot Score | Mr (Da) | Coverage (%) | Protein p*I* |
| gi\|456358995 | P2 | 26(8) | 749* | 148426 | 26 | 6,36 |
|  |  | R.AWLHVGLNMAK.H |  | 1239 |  |  |
|  |  | R.GFAFGVLSR.M | 43 | 953 |  |  |
|  |  | R.LQELGENDASTADTYLTWELACAHGK.G |  | 2892 |  |  |
|  |  | K.GEVAITPVPAAWLDPEAQLTGR.E | 32 | 2290 |  |  |
|  |  | K.IDGVTQNAAENAR.V |  | 1358 |  |  |
|  |  | R.VHYATRPDPMSWLDDNTGLSADSNAGR.I |  | 2945 |  |  |
|  |  | R.ISGEHYTLWK.G |  | 1233 |  |  |
|  |  | K.GFEGLGACAEFLLANSR.F | 96 | 1811 |  |  |
|  |  | K.AQLATATYALMR.R + Oxidation (M) |  | 1325 |  |  |
|  |  | R.RYDISER.T |  | 937 |  |  |
|  |  | R.TCHFAITTIGHMVAQTAVR.D |  | 2113 |  |  |
|  |  | R.DLNNGSLSPLPFR.V | 83 | 1429 |  |  |
|  |  | R.FTDVHTVATDLFR.K | 94 | 1521 |  |  |
|  |  | R.GSEVTHSSQEALFAQK.M |  | 1718 |  |  |
|  |  | K.AVWSSMAEGSTR.L |  | 1281 |  |  |
|  |  | R.LYNLNQAYGPFVDVQLAR.I | 133 | 2080 |  |  |
|  |  | R.WFATTRLNAAGSK.V |  | 1422 |  |  |
|  |  | R.WTNGLNSDDR.A |  | 1177 |  |  |
|  |  | R.AGVHVFAYGR.S | 61 | 1076 |  |  |
|  |  | R.TANIVAGAVLGAR.N |  | 1212 |  |  |
|  |  | R.MSTSPEAHRR.G |  | 1171 |  |  |
|  |  | R.GVLASDYHVDIATDGNIR.H |  | 1915 |  |  |
|  |  | R.EVYTVADIPTVSER.V |  | 1578 |  |  |
|  |  | R.VSGLALRPYER.S | 22 | 1260 |  |  |
|  |  | K.LGDNTPVTNR.Q |  | 1086 |  |  |
|  |  | R.QALRPPEYNR.N |  | 1243 |  |  |
|  |  |  |  |  |  |  |
| gi\|456358999 | P4 | 36(3) | **724*** | 113986 | 40 | 6,18 |
|  |  | R.SPTSVGNTVAADVQTSVHDKPTGELK.G |  | 2637 |  |  |
|  |  | K.GSDGTGIHEATGLPIDKR.G |  | 1823 |  |  |
|  |  | K.RGEVPTVQLER.T |  | 1283 |  |  |
|  |  | R.GEVPTVQLER.T |  | 1127 |  |  |
|  |  | K.LMLQQEFQNIVACAK.N + Oxidation (M) |  | 1808 |  |  |
|  |  | K.NAPQMTVNAGR.F |  | 1158 |  |  |
|  |  | K.RIEGQCVVALEPLTITLSGSTSSTQDNSDSAK.L |  | 3364 |  |  |
|  |  | R.SRDGWLDHQTDFAVK.G |  | 1774 |  |  |
|  |  | R.DGWLDHQTDFAVK.G |  | 1531 |  |  |
|  |  | R.MVVHSTLR.K |  | 942 |  |  |
|  |  | K.CDAGVAAAALALTWGKPK.L |  | 1799 |  |  |
|  |  | K.LGGAGHANLTAVMSEAGVGYITGVNGTR.A | 41 | 2672 |  |  |
|  |  | R.ATPHADTVFGR.E |  | 1171 |  |  |
|  |  | R.ATPHADTVFGREELVYLLGFALR.H |  | 2574 |  |  |
|  |  | R.EELVYLLGFALR.H | 103 | 1422 |  |  |
|  |  | R.HMADAQEQVIR.N |  | 1297 |  |  |
|  |  | R.NVLAQVASLFR.P | 79 | 1217 |  |  |
|  |  | K.VSRPMNEPAFR.E |  | 1303 |  |  |
|  |  | R.PMNEPAFR.E |  | 960 |  |  |
|  |  | R.EVWNVANSSSDLQMIDR.D |  | 1963 |  |  |
|  |  | R.EVWNVANSSSDLQMIDRDK.L |  | 2206 |  |  |
|  |  | R.DKLNGEHFLR.Q |  | 1228 |  |  |
|  |  | K.DLYHHLFQYATTTYADGVQVMQANTR.M |  | 3042 |  |  |
|  |  | R.EVYNWLLWHACK.T |  | 1618 |  |  |
|  |  | K.SAVEWLSSNSVEAHR.F | 81 | 1671 |  |  |
|  |  | R.SSAGLGATEAAGSPGR.R |  | 1388 |  |  |
|  |  | R.LHHYDGQIFSNVIADTER.H | 105 | 2114 |  |  |
|  |  | R.RLYTPSELR.D |  | 1134 |  |  |
|  |  | R.LYTPSELR.D |  | 978 |  |  |
|  |  | R.NDLFVVDR.I |  | 977 |  |  |
|  |  | R.AQLMLISVQEDGGR.H | 59 | 1516 |  |  |
|  |  | R.HGIEAIIDTNGSVSVK.V |  | 1639 |  |  |
|  |  | R.FCLYDDNK.T |  | 1073 |  |  |
|  |  | R.FCLYDDNKTSSYITAQESR.T |  | 2297 |  |  |
|  |  | K.TSSYITAQESR.T |  | 242 |  |  |
|  |  | K.NANTISGLVAHDYK.L |  | 1502 |  |  |

*Protein Score
